# Patient-reported physical activity questionnaires: A systematic review of content and format

**DOI:** 10.1186/1477-7525-10-28

**Published:** 2012-03-13

**Authors:** Kate Williams, Anja Frei, Anders Vetsch, Fabienne Dobbels, Milo A Puhan, Katja Rüdell

**Affiliations:** 1 Patient Reported Outcomes Centre of Excellence, Global Market Access, Primary Care Business Unit, Pfizer Ltd, Walton Oaks, Surrey, UK; 2 Horten Centre for Patient-oriented Research, University Hospital of Zurich, Zurich, Switzerland; 3 Institute of General Practice & Health Services Research, University Hospital of Zurich, Zurich, Switzerland; 4 Centre for Health Services & Nursing Research, Katholieke Universiteit Leuven, Leuven, Belgium; 5 Department of Epidemiology, Johns Hopkins Bloomberg School of Public Health, Baltimore, USA

**Keywords:** Physical activity, Chronic illness, Patient-reported outcome questionnaires, Systematic review

## Abstract

**Background:**

Many patients with chronic illness are limited in their physical activities. This systematic review evaluates the content and format of patient-reported outcome (PRO) questionnaires that measure physical activity in elderly and chronically ill populations.

**Methods:**

Questionnaires were identified by a systematic literature search of electronic databases (Medline, Embase, PsychINFO & CINAHL), hand searches (reference sections and PROQOLID database) and expert input. A qualitative analysis was conducted to assess the content and format of the questionnaires and a Venn diagram was produced to illustrate this. Each stage of the review process was conducted by at least two independent reviewers.

**Results:**

104 questionnaires fulfilled our criteria. From these, 182 physical activity domains and 1965 items were extracted. Initial qualitative analysis of the domains found 11 categories. Further synthesis of the domains found 4 broad categories: 'physical activity related to general activities and mobility', 'physical activity related to activities of daily living', 'physical activity related to work, social or leisure time activities', and '(disease-specific) symptoms related to physical activity'. The Venn diagram showed that no questionnaires covered all 4 categories and that the '(disease-specific) symptoms related to physical activity' category was often not combined with the other categories.

**Conclusions:**

A large number of questionnaires with a broad range of physical activity content were identified. Although the content could be broadly organised, there was no consensus on the content and format of physical activity PRO questionnaires in elderly and chronically ill populations. Nevertheless, this systematic review will help investigators to select a physical activity PRO questionnaire that best serves their research question and context.

## Background

Many patients with chronic diseases experience physical activity limitations or suffer symptoms during physical activities. This is concerning given the wealth of evidence demonstrating the importance of a physically active lifestyle in the prevention and management of many chronic diseases [1,2]. Physical activity has been defined as 'any bodily movement produced by the contraction of skeletal muscle that increases energy expenditure above a basal level' [3]. It is useful as an outcome measurement as it enables researchers to effectively evaluate public health interventions to increase physical activity levels. It is also currently being explored as an endpoint for evaluating the efficacy of pharmaceutical interventions in clinical trials. This could help inform patients about treatment options that may improve their daily life.

When deciding to assess physical activity as an outcome measure, researchers face the challenge of selecting from a myriad of objective and subjective assessments. For subjective assessments, a large number of patient-reported outcome (PRO) questionnaires are available to choose from. PRO questionnaires are self-report measures of a patient's health status or behaviour that comes directly from the patient without interpretation from anyone else. Such questionnaires have the potential to capture patient-relevant lifestyle physical activities and related limitations that may not be identified by more objective assessments. For this reason, it is important that the content of physical activity PRO questionnaires is relevant to the patient in order to make appropriate, patient centred treatment choices [4,5]. In addition, the format of the questionnaire should be such that the questions and answer options can be easily interpreted and completed by the patient.

Although there have been several reviews of physical activity PRO questionnaires in recent years (e.g. [6]), the majority of these have focused on the development and validation processes. To our knowledge, no review to date has specifically focused in depth on content and format such as looking at themes and patterns across questionnaires.

This review is part of the European Union funded PROactive project [7] which aims to develop and validate a PRO tool to investigate dimensions of physical activity in chronic obstructive pulmonary disease (COPD) patients. The initial aim of this review was therefore to identify existing physical activity PRO questionnaires which are appropriate for use in a COPD population. Although we were primarily interested in questionnaires developed for COPD patients, we were also interested in learning from questionnaires developed for elderly populations or patients with other chronic diseases that may result in physical activity limitations. The second aim was to systematically evaluate these questionnaires with the aim of establishing if there is a consensus on their optimal content and format (the development and psychometric properties are explored in a separate paper [8]). These results may help researchers to select the most appropriate physical activity PRO questionnaires available to date, and will identify research gaps.

## Methods

A study protocol (unregistered) guided the entire review process. We followed standard systematic review methodology as outlined in the handbooks of the Centre for Reviews and Dissemination [9] and the Cochrane Collaboration. The reporting follows the PRISMA statement guidelines for reporting systematic reviews and meta-analyses [10].

### Eligibility criteria

#### Population

As this systematic review is part of the PROactive project [7], we were interested in identifying physical activity PRO questionnaires that are appropriate for use in a COPD population. We therefore supplemented the electronic database search with explicit search terms for COPD patients. However, we were also interested in learning from the content and format of questionnaires developed for other disease populations which may experience similar physical activity limitations to COPD patients. We therefore expanded our search to include PRO questionnaires developed for patients with all chronic illnesses and elderly populations.

#### Style of questionnaire

We included fully structured questionnaires or scales with standardised questions and answer options which were patient (self) reported. Interviewer administered questionnaires were included only if the information was self-reported. Questionnaires that required a rating by an interviewer were excluded.

#### Assessment of physical activity

We included questionnaires containing at least one physical activity subscale/domain. We used this benchmark as the number of questionnaires containing only one or two physical activity items was too large to include in this review. The PROactive consortium agreed to use the following definition for physical activity by the U.S. Department of Health and Human Services [3]: 'any bodily movement produced by the contraction of skeletal muscle that increases energy expenditure above a basal level'. In addition to questionnaires measuring the frequency, intensity and total amount of physical activity, we also considered questionnaires assessing 'related constructs' such as symptoms (physical and mental) or limitations associated with physical activity. We only included questionnaires if the items were available from the publication or developers. We did not have any language or publication date restrictions.

#### Study design

We included cross-sectional and longitudinal studies that described the development or modifications of the original questionnaire and/or the initial validation of the original questionnaire. We excluded studies that were not designed to initially validate a questionnaire, for example, those that reported linguistic validation or used a questionnaire as an outcome measure in a clinical trial or observational study.

### Information sources

#### Electronic database searches

We searched the electronic databases Medline, Embase, PsycINFO and CINAHL on September 18th 2009.

#### Hand searches

In addition to the electronic database search, we did the following hand searches: we searched for original development studies of questionnaires from articles which were excluded for the reason 'validation only' or 'used as outcome measures'; we scanned the reference lists of the full texts; we searched for 'physical functioning' questionnaires in the Patient-Reported Outcome and Quality of Life Questionnaires Database (PROQOLID) on March 10 2010; and we contacted experts in the field (the PROactive research consortium and associated expert panel) to check that our list was complete.

### Search

We searched the electronic databases using the following search terms: (physical activity OR functioning OR function OR motor activity OR activities of daily living OR walking OR activity OR exercise) AND (questionnaire* OR scale OR tool OR diary OR assessment OR self-report OR measure*) AND (valid*) AND (chronic disease OR elderly OR COPD OR chronic lung disease OR chronic obstructive lung disease) NOT (athletic performance OR sports OR children OR adolescent).

### Study selection

The study selection process was piloted by at least two independent reviewers at the start of the review. All titles and abstracts were screened and the decision to include or exclude was recorded (0 = exclude, 1 = order for full text assessment, 2 = only validation study of existing questionnaire, 3 = related study (e.g. reviews), do not order but may be useful reference). All articles that were deemed potentially eligible by at least one reviewer proceeded to full text review. The full texts were then scored against the predefined selection criteria and the decision to include or exclude was again recorded. If there was a discrepancy between two reviewers, a third reviewer was consulted. If the article contained insufficient information then we made three attempts to contact the authors and recorded the outcome. In cases where multiple papers were published (e.g. translations, reporting on different outcomes etc.), we treated the multiple reports as a single study but made reference to all publications.

### Data extraction process

We created standardised data extraction forms to record the relevant information from the articles. The data extraction forms were piloted twice by four reviewers. The forms and categories were then adapted and refined where necessary. The first reviewers extracted the data and stored it in a MS Word file. The second reviewers then independently extracted the data and compared their results with that of the first reviewers. Discrepancies were resolved by consulting a third independent reviewer.

### Data extraction

We extracted data on the questionnaires' content and format. The format categories were: population (elderly or type of chronic disease), answer options (e.g. 5-point Likert scale, categorical scales), anchors (e.g. 0 = not limited at all to 6 = totally limited), scoring (e.g. total score or average), direction of scale (uni- or bi-directional), recall period (e.g. past 24 hours or past week), administration (self or interviewer administered), quantification (whether questionnaires quantified the amount of physical activity [e.g. number of hours spent] or not), and type of questionnaire (quick overview of the method of assessment [e.g. ability, frequency], the content of assessment [e.g. breathlessness] and the population). The content categories were: a general description of the questionnaire (physical activity only or general questionnaire with physical activity subscales), number of items, number of domains, and labelling of domains.

### Content analysis

Content analysis of the domain labels was conducted to synthesise the data. The domains were independently grouped into broad categories by two reviewers and their level of agreement was calculated using Cohen's Kappa coefficient. Mismatches were then resolved and a third reviewer was consulted where necessary. Once the categorisation of all the domains had been agreed, the frequency of domains per category was calculated. Following the categorisation of domains into broad categories, a second content analysis was conducted to further synthesise the content of the questionnaires. This was again done by two independent reviewers and a third reviewer was consulted where necessary. A Venn diagram was then produced to give a visual representation of the content of the questionnaires. A brief content analysis was also conducted for the populations for which the questionnaires were developed (focusing on COPD and related respiratory diseases) and the answer options used.

## Results

### Study selection

**Figure 1:**
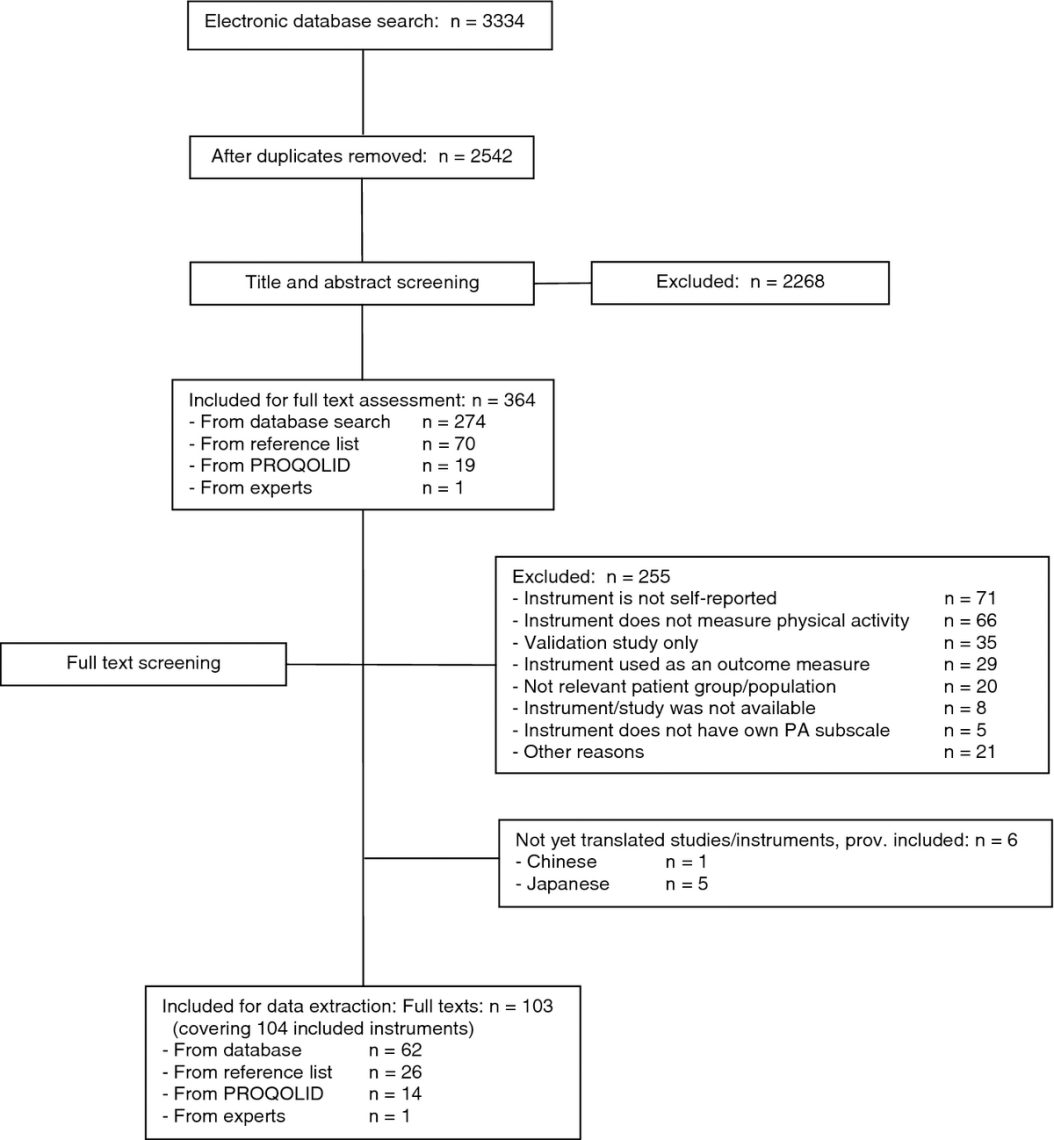
**Flow diagram showing the study identification process**. The diagram shows the process we followed to identify relevant studies and the number of studies that were included or excluded at each stage

Figure 1 shows a flow diagram of the study identification process. The electronic database search produced 2542 references. After title and abstract screening, 2268 of these were excluded resulting in 274 for full text assessment. This included 5 Japanese and 1 Chinese article which were provisionally included due to their English abstract but were not included in the current analysis as we were unable to translate them [11-15]. Hand searches of reference sections and of excluded articles revealed an additional 70 questionnaires/development studies for full text assessment. The search of the PROQOLID database produced a further 58 questionnaires, 19 of which were included for full text assessment after title and abstract screening. One additional questionnaire was retrieved from the consultation with experts. Therefore, a total of 364 papers were included for full text assessment.

Following full text assessment, a further 255 articles were excluded resulting in 104 questionnaires from 103 full texts included in the review [16-119] (one article [65] provided information for the development process of two questionnaires). The most frequent reasons for exclusion were: the questionnaire is not self-reported (n = 71), the questionnaire does not measure physical activity (defined as above [3]) (n = 66), the article was a validation study only (other than the original validation) (n = 35) and the article used the questionnaire as an outcome measure only (did not describe the development or initial validation) (n = 29). The references of all articles excluded after full text assessment are summarised in Additional file 1.

### Content of questionnaires

Additional file 2 summarises the extracted data on the content and format of the reviewed questionnaires.

Fifty nine (56.7%) questionnaires focused on physical activity only. Forty three (41.3%) did not focus on physical activity but contained at least one physical activity subscale. Two (1.9%) questionnaires [78,118] were not described in the publication and the questionnaires were not available from the authors.

A total of 1965 items (a further 5 questionnaires did not report the number of items) relating to physical activity were extracted. The items were not checked for duplicates due to their large number; however, it is unlikely that the items with exactly the same wording would have appeared multiple times. The number of physical activity items per questionnaire ranged from 3 to 123.

After the removal of 56 duplicate domains, a total of 182 physical activity domains (a further 2 articles did not report their domains) were extracted. The number of physical activity domains per questionnaire ranged from 1 to 12. The domains that appeared multiple times are shown in Table 1. All other domains appeared only once.

**Table 1:** Physical activity domains (as described by the authors) that appeared multiple times

Physical activity domain	N	Physical activity domain	N
Activities of daily living/ADL*	10	Falls efficacy	2

Mobility	8	Ambulation	2

Leisure activities	6	Domestic tasks	2

Physical activity(ies)	8	Domestic chores	2

Physical function(ing)	7	Family role	2

Self-care	4	Social functioning	2

Activity	3	Care taking	2

Exercise	3	Work	2

Household activities	3	Disability	2

Instrumental activities of daily living/IADL**	2		

*ADL = Activities of daily living

**IADL = Instrumental activities of daily living

The initial thematic analysis of the 182 physical activity domains found 11 broad categories, plus an additional 'other' category (defined in Table 2). The inter-rater reliability of the initial independent coding of the 182 domains was high with a Cohen's Kappa of 0.87 (p < 0.001) and 88.5% total accordance. After agreement for mismatches, the number of the domains per content theme were: physical activity related mobility (n = 34), household physical activity (n = 21), generic physical activity (n = 20), social physical activity (n = 18), physical activity relating to self (n = 17), dyspnoea & symptom related physical activity (n = 12), leisure physical activity (n = 9), work physical activity (n = 9), exercise physical activity (n = 10), physical activity limitations (n = 8), activities of daily living (ADL) (n = 7) and other (n = 17). The full list of domains and their 11 categories are shown in Additional file 2.

**Table 2:** Eleven categories identified from the initial content analysis of the physical activity domains

Category	Definition
Generic physical activity	Domains that relate to physical activity/functioning in general that do not specify a particular type of physical activity.

Activities of daily living (ADL)	Domains referring specifically to activities of daily living or instrumental activities of daily living.

Dyspnoea and symptom related physical activity	Domains that refer to dyspnoea and/or other symptoms which may occur as a result of physical activity.

Exercise physical activity	Domains referring to exercise or other activities that are more vigorous than usual everyday activities.

Physical activity relating to self	Domains referring to a person's ability to look after themselves. Also includes domains about their belief that they can look after themselves and other self beliefs.

Physical activity related mobility	Domains referring to body movement or a person's ability to move around both inside and outside their home.

Leisure physical activity	Domains referring to leisure or recreational activities. These are not necessarily activities that are done socially but include activities that can be done alone.

Household physical activity	Includes all domains referring to activities within the home and/or garden.

Social physical activity	Domains referring to social activities including those involving friends, family, community and intimate relationships.

Work physical activity	Domains referring to paid or unpaid work or education.

Physical activity limitations	Domains referring to physical activity limitations or disability (likely to be due to a physical condition such as COPD).

Other	Any other domains which do not fit into the other categories.

**Figure 2:**
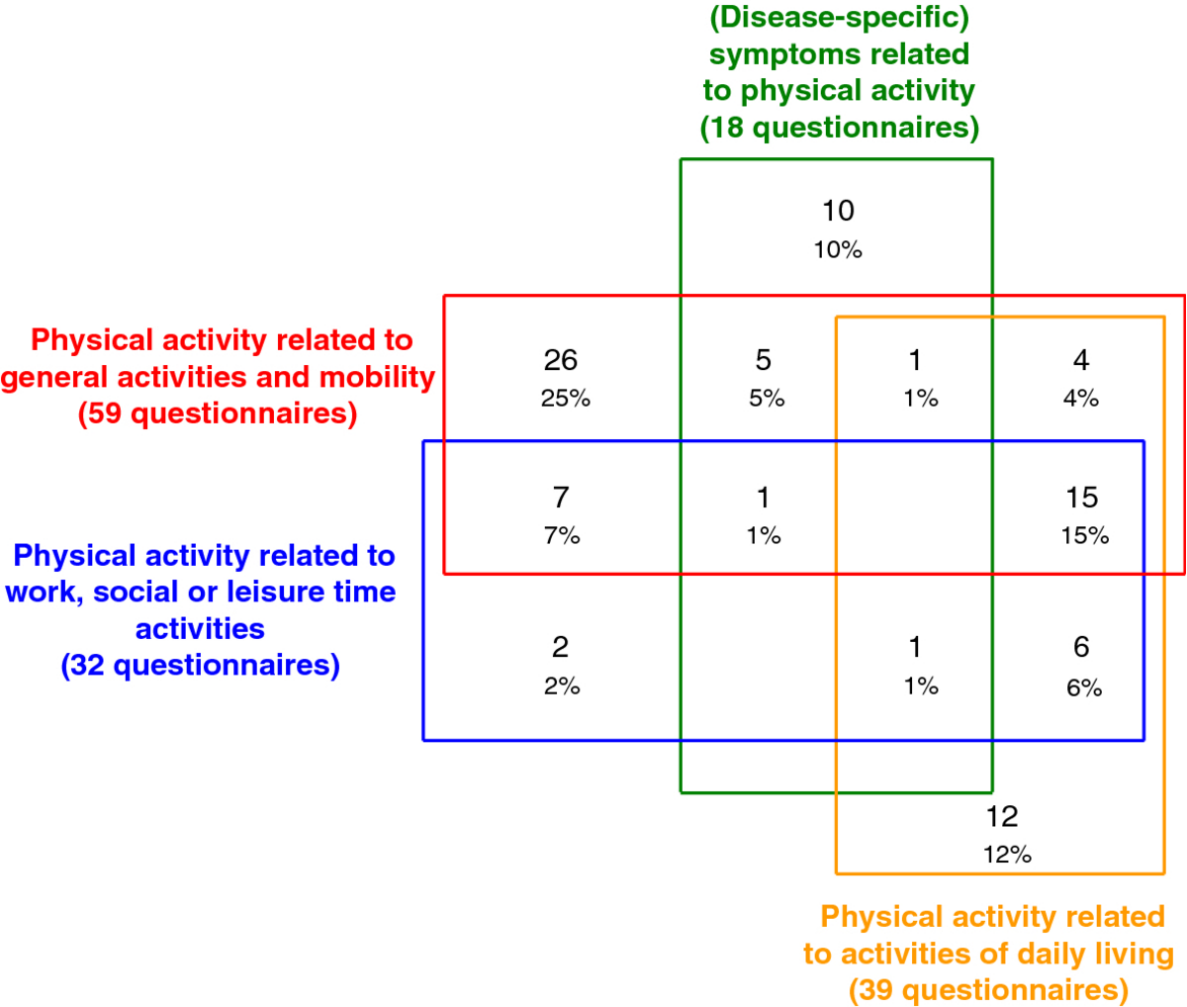
**Venn diagram showing overlapping categories of domains**. The Venn diagram shows how the four categories of domains overlap with each other and the percentage of questionnaires that include domains in each category

The second content analysis resulted in 4 categories plus an additional 'other' category (defined in Table 3). The Venn diagram in Figure 2 illustrates the distribution of the questionnaires across these 4 categories. This shows that 59 questionnaires contained the domain 'physical activity related to general activities and mobility', 39 the domain 'physical activity related to activities of daily living', 32 the domain 'physical activity related to work, social or leisure time activities', and 18 the domain '(disease-specific) symptoms related to physical activity'. The Venn diagram also shows that none of the questionnaires contained domains from all 4 of the categories. Further, questionnaires containing '(disease-specific) symptoms related to physical activity' domains did not often contain domains from the other 3 categories as well.

**Table 3:** Four categories identified from the second content analysis of the physical activity domains

Categories	Definition
Physical activity related to general activities and mobility	Domains that relate to physical activity and functioning in general and a person's ability to move around that do not specify a specific type of physical activity. This category also includes physical exercise or other general activities that are more vigorous than usual everyday activities.

Physical activity related to activities of daily living	Domains referring specifically to activities of daily living (ADL, such as eating, toileting, bathing, dressing) or to instrumental activities of daily living (IADL, such as shopping, use of transportation, housekeeping, food preparation).

Physical activity related to work, social or leisure time activities	Domains referring to social activities, to paid or unpaid work or education and to leisure or recreational activities.

(Disease-specific) symptom related to physical activity	Domains that refer to dyspnoea and/or other symptoms which may occur as a result of physical activity and domains referring to physical activity limitations, disability or difficulties an individual may have in executing activities.

Other	Any other domains which do not fit into the other categories.

### Format of questionnaires

The questionnaires were developed for patients with a range of chronic diseases and elderly populations. These populations were grouped into the 5 categories 'Elderly', 'COPD patients', 'Patients with other chronic respiratory diseases', 'Patients with unspecified chronic disease or disability', and 'Patients with other specified chronic diseases' (Table 4).

**Table 4:** Categorisation of the populations for which the included questionnaires were developed (n = 104)

Population	N	%
**Elderly**	32	30.8%

**COPD patients**	15	14.4%

**Patients with other chronic respiratory diseases**	12	11.5%
(Chronic respiratory failure, (unspecified) chronic lung disease, chronic airflow limitation, asthma, (unspecified) pulmonary impairment, patients receiving home mechanical ventilation, and various underlying diseases)		

**Patients with unspecified chronic disease or disability**	15	14.4%

**Patients with other specified chronic diseases**	30	28.8
(Chronic pain, multiple sclerosis, stroke, heart failure, coronary heart disease, peripheral arterial disease, chronic liver disease, minimal hepatic encephalopathy, cancer, back pain, chronic fatigue syndrome, immune thrombocytopenic purpura, chronic urticaria, women with fibromyalgia, ankylosing spondylitis, chronic disabling musculoskeletal disorders, osteoarthritis, rheumatoid arthritis, (unspecified) rheumatoid diseases, cardiovascular disease and osteoporosis)		

Analysis of the 1965 items revealed 12 different types of answer option (Table 5) and 209 different anchors (duplicate anchors removed). The full list can be seen in Additional file 2. Of the 209 different anchors, the most frequent was the categorical yes/no scale which was used for 265 items overall.

**Table 5:** Answer options and the frequency of their occurrence

Answer options	Number of different types
3-point scales	40

5-point Likert scales	40

Categorical scales (defined categories to select e.g. yes/no)	37

4-point scales	36

Frequency/duration (e.g. number of times per week or number of hours spent)	14

7-point Likert type scales	11

Visual analogue scale (VAS)	11

6-point Likert type scales	8

11-point Likert type scales	6

10-point Likert type scales	3

Free report	2

Diary	1

Sixty eight (65.4%) questionnaires were scored by calculating the sum of the items to domains scores and total scores, 10 (9.6%) by calculating a mean score of completed items, 5 (4.8%) using Guttman scaling and 6 (5.8%) using another method classified as 'other'. Fifteen (14.4%) questionnaires did not report the method of scoring used.

Seventy three (70.2%) questionnaires were uni-directional, meaning that the items were phrased in the same direction, either positively or negatively. Three questionnaires (2.9%) were bi-directional, 1 (1%) contained uni-directional and bi-directional items and 27 (26%) did not report the scale direction or direction was not applicable (e.g. categorical scales).

Forty two different recall periods were identified and these were grouped thematically into 10 categories plus a 'not reported/unclear' category. Table 6 shows the categories along with the number of questionnaires to which they apply.

**Table 6:** Categorisation of recall periods and the frequency of their occurrence

Recall period	N	%
No recall period	31	29.8%

Present/today	6	5.8%

Yesterday/past few days	4	3.8%

Past week	15	14.4%

Past 2 weeks	8	7.7%

Past month	10	9.6%

Past 3 months	1	1%

Past year	1	1%

Multiple different recall periods	5	4.8%

General	1	1%

Not reported/unclear	22	21.2%

Fifty eight (55.8%) questionnaires were self-administered, 25 (24%) were interviewer-administered and 16 (15.4%) were either self- or interviewer-administered. Five (4.8%) questionnaires did not report their administration format.

Nine of the questionnaires quantified the amount of physical activity engaged in (e.g. total time, duration), whereas the other 95 did not. These questionnaires can be seen in row 3 of Table 7.

**Table 7:** Frequency of each 'type' of questionnaire

Method and content of assessment	Frequency	Reference number(s)
Ability/capacity to perform physical activities*	25	[21,25-30,37,41,42,45,47,48,53,54,70,72,82,86,89,91,97,99,100,113,114]

Frequency/categorised amount of time performing physical activities (no quantification of physical activities)	13	[19,32,40,56,61,67,65]a** [87,93,103,108,109,116]

Quantification of physical activities: Total time/duration/diary	9	[30,36,44,46,74,65]b** [102,110,119]

Degree/level/frequency of limitations/symptoms/difficulty in performing physical activities	35	[20,23,33,38,39,49,50,55,58,60,64,67-69,73,75-77,79-81,84,85,87,90,94-96,99-101,105,107,111,115,117]

Impact of symptoms/disease/functional impairment on physical activities	7	[16,17,22,52,57,66,106]

Self-efficacy/confidence in performing physical activities	7	[18,24,35,80,92,104,112]

Degree of dependence/independence	5	[43,59,63,88,98]

Graduation of needed help/amount of assistance needed in performing physical activities	2	[51,62]

Excluded from categorisation because we did not have access to the full original questionnaires and therefore had too little information to categorise them.		[31,34,71,78,83,118]

*Physical activity includes all kind of activities

**[65]a = LTPAI, [65]b = PAHWI (see reference)

X allocated to more than one questionnaire type

We identified 8 types of questionnaire based on their method of assessing physical activity (e.g. ability) and the content of this assessment (e.g. limitations). These types, along with the frequency of questionnaire for each type and the reference numbers of the questionnaires for each type are shown in Table 7.

## Discussion

This systematic review found many PRO questionnaires for assessing physical activity. Most questionnaires focused on physical activity alone (see definition [3]) but there were also multiple questionnaires containing physical activity domains or subscales. Most questionnaires were developed for patients with chronic diseases, although the single largest group was elderly. The format of the questionnaires including the answer options, anchors and recall periods varied considerably. The most common answer option was the yes/no scale. Most questionnaires had no recall period, were uni-directional, self-administered and scored by calculating the sum of the domain or total scores.

Multiple domains and items were extracted and although the domains were grouped broadly into 11 categories, the content varied considerably. Further synthesis into 4 categories and the Venn diagram revealed that no questionnaires contained domains from all 4 categories. This was surprising as we expected to see increased overlap due to the large number of domains and the small number of categories. However, we acknowledge that the questionnaires were developed for a range of populations and limitations experienced by some groups may not be universal. The Venn diagram also showed that '(disease-specific) symptoms related to physical activity' were included by the fewest questionnaires and infrequently overlapped with the other categories. This shows that symptoms and limitations related to physical activity are not prominent in the currently available PRO questionnaires. This is concerning as qualitative research has shown that patients with certain chronic conditions (e.g. asthma) consider symptoms in association with physical activity to be very relevant [120]. This inconsistency may be due to inadequate patient input in the development of these questionnaires as was found in the first part of this review [8]. However, we acknowledge that symptoms are not a relevant aspect of all chronic conditions (e.g. hypertension).

Overall the results show that there is no consensus on what should be included in the content and format of physical activity PRO questionnaires. This is in line with previous reviews which have found variation in the number of recall periods used [6] and inconsistencies in the development and validation methods questionnaires [6,8]. The lack of consensus may also arise from the scarcity of conceptual frameworks for physical activity, which was documented recently [121]. This highlights a need for further research into physical activity and its potential use as an outcome measure to evaluate treatment benefit. In addition, the results show that many physical activity questionnaires lack important concepts, particularly those relating to symptoms and limitations with physical activity. This poses a problem to researchers when deciding which physical activity PRO questionnaire to choose for their purpose as no questionnaire measures all aspects of physical activity. Although this highlights a need for patient input in the development of future physical activity questionnaires, it is also important to acknowledge that physical activity is a multidimensional construct. It is therefore challenging to create a single questionnaire which encompasses all aspects.

Nevertheless, both this review and our previous systematic review [8] provide a broad overview of physical activity questionnaires and can be used to guide researchers in their selection a questionnaire. For example, a questionnaire may be needed to assess physical activity as an outcome in a pulmonary rehabilitation intervention study of COPD patients (example 1). As another example, investigators may need a questionnaire to assess the association between physical activity and mortality in a prospective cohort study of elderly people (example 2). In situations like these, Additional file 2 will be a useful tool for researchers as it summarises the content and format of the large variety of available questionnaires.

To evaluate pulmonary rehabilitation (as in example 1), a suitable questionnaire may be one that was specifically developed for COPD patients (see 'Population' in Additional file 2) and that assesses domains that a pulmonary rehabilitation program aims to improve (e.g. the patients' ability to perform activities of daily living, see 'Questionnaire type'). Even more specifically, investigators could choose between different types of activities of daily living or household physical activities (see 'Category' and 'Labelling of domains'). Since a study on pulmonary rehabilitation is typically designed to detect a change over time, a unidirectional Likert type scale would be reasonable, encompassing at least 5 points, with corresponding anchors (see 'Direction of scale', 'Answer options' and 'Anchors') resulting in different domain and total scores (see 'Scoring'). Depending on the number of other assessments they may be using, investigators may also want to consider the time to complete ('Number of items') and the recall period ('Recall period') in order to minimise information bias. Based on these considerations, the London Chest Activity of Daily Living Scale [49] or the Activity of Daily Living Dyspnoea scale [115] would be reasonable choices.

If physical activity is measured as a determinant of mortality (as in example 2), the amount of physical activity ('Quantification', 'Questionnaire type') is likely to be of importance (e.g. [122]) and could be expressed by the frequency and time spent for performing certain activities ('Category', 'Labelling of domains'). A single number representing the amount of physical activity ('Scoring') would be attractive from a statistical and interpretative perspective. Also, as the researcher may be assessing other determinants of mortality, the length of the questionnaire should be considered to avoid patient burden ('Number of items'). An appropriate questionnaire for this example would be the YALE Physical Activity Survey [36].

During the selection process, the measurement properties also need to be considered once potential questionnaires have been identified based on content and format requirements. For an overview of the development and initial validation data of the questionnaires, readers are referred to Additional file 2 in our previous publication [8].

One of the strengths of this review is that we adhered to a rigorous systematic review methodology throughout the process. We used carefully developed inclusion and exclusion criteria and each step was conducted by at least two independent reviewers from at least two independent institutions to ensure that the most appropriate physical activity questionnaires were included. We kept our search strategy deliberately broad to avoid missing any potentially relevant questionnaires, resulting in what is likely to be the most comprehensive systematic review of physical activity questionnaires to date. We did this by using the definition for physical activity as described in the 2008 physical activity guidelines for Americans [3] as a guide. In addition to public database searches we added a thorough hand search of reference sections and the PROQOLID database, resulting in an extensive domain and item pool of physical activity questionnaires.

A challenge of this review was dealing with situations where the decision to include or exclude a questionnaire was unclear. Although we followed carefully defined inclusion and exclusion criteria, some questionnaires assessed specific types of physical activity that were largely unique to the population for which they were developed. In such cases we attempted to make a judgement to include or exclude that was systematically and scientifically defendable. For example, if a questionnaire had been developed for multiple sclerosis patients, we excluded physical activity domains that assessed impaired hand motor activity, but included general domains such as 'walking ability' [55] or 'physical functioning' [95]. Furthermore, although we did not analyse the content of the individual items, they were all entered into an item pool which can be utilised during the later stages of the PROactive project and will be made available to the public upon the conclusion of the project.

## Conclusions

This review found a large number of PRO questionnaires are available for assessing physical activity in elderly and chronically ill populations. From these, 182 different physical activity domains were identified. Although the content could be broadly organised, there was little consensus on the content and format of physical activity PRO questionnaires in these populations. Nevertheless, this systematic review will help investigators to select a physical activity PRO questionnaire that best serves their research question and context.

## Abbreviations

PRO: Patient-reported outcome; ADL: Activities of daily living; IADL: Instrumental activities of daily living.

## Competing interests

Kate Williams was contracted by Pfizer Ltd when this review was conducted and Katja Rüdell is an employee of Pfizer Ltd. The current work is methodological and provides no competitive advantage or disadvantage. Anja Frei, Anders Vetsch, Fabienne Dobbels and Milo Puhan have no competing interests. No medical writers were used to support the writing of this manuscript.

## Authors' contributions

KR and MP led the systematic review. KR, MP, AF and FD developed the conceptual framework and the study protocol. KR, MP and AF coordinated the review. KR, MP, FD, and AF conducted the electronic database searches; KW, AV, AF, KR and MP conducted the additional searches. AF coordinated the references in RefWorks. KR and FD (1^st ^reviewers), KW (2^nd ^reviewer) and MP and AF (3^rd ^reviewers) screened titles and abstracts. KW and KR (1^st ^reviewers), AF and AV (2^nd ^reviewers) and MP (3^rd ^reviewer) assessed full texts of the identified studies. KW and AV (1^st ^reviewers) and KR and AF (2^nd ^reviewers) extracted the relevant data. KW conducted the statistical analysis. KW drafted the manuscript. All authors contributed to revising the manuscript and approved the final version.

## Authors' information

KR is an honorary lecturer of health psychology at the University of Kent, UK. Fabienne Dobbels is a post-doctoral researcher funded by the FWO (Scientific Research Foundation Flanders).

## Acknowledgements

The study was conducted within the PROactive project which is funded by the Innovative Medicines Initiative Joint Undertaking (IMI JU) # 115011. The authors would also like to thank Laura Jacobs for her support in the early stages of the project as well as the PROactive group: Caterina Brindicci and Tim Higenbottam (Chiesi Farmaceutici S.A.), Thierry Trooster and Fabienne Dobbels (Katholieke Universiteit Leuven), Margaret X. Tabberer (Glaxo Smith Kline), Roberto Rabinovitch and Bill McNee (University of Edinburgh, Old College South Bridge), Ioannis Vogiatzis (Thorax Research Foundation, Athens), Michael Polkey and Nick Hopkinson (Royal Brompton and Harefield NHS Foundation Trust), Judith Garcia-Aymerich (Municipal Institute of Medical Research, Barcelona), Milo Puhan and Anja Frei (Universität of Zürich, Zürich), Thys van der Molen and Corina De Jong (University Medical Center, Groningen), Pim de Boer (Netherlands Asthma Foundation, Leusden), Ian Jarrod (British Lung Foundation, UK), Paul McBride (Choice Healthcare Solution, UK), Nadia Kamel (European Respiratory Society, Lausanne), Katja Rudell and Frederick J. Wilson (Pfizer Ltd), Nathalie Ivanoff (Almirall), Karoly Kulich and Alistair Glendenning (Novartis), Niklas X. Karlsson and Solange Corriol-Rohou (AstraZeneca AB), Enkeleida Nikai (UCB) and Damijen Erzen (Boehringer Ingelheim).

## Supplementary Material

Additional file 1 **Reference list of excluded articles after full text assessment**. List of all references of articles which were excluded after full text assessment [123-377]. Click here for file

Additional file 2 **Data extraction results: content and format of the reviewed questionnaires**. Summary of the extracted data on the content and format of the reviewed questionnaires. This covers the population, domains, categories, items, answer options, anchors, scoring, direction of scale, recall period, administration, questionnaire type and quantification. Click here for file
