# Supplementary material for: Patient-reported physical activity questionnaires: A systematic review of content and format
Source: Health Qual Life Outcomes. 2012 Mar 13;10:28. doi: 10.1186/1477-7525-10-28 (PMC3349541; doi:10.1186/1477-7525-10-28)
Supplement: Additional file 2 — Data extraction results: content and format of the reviewed questionnaires. Summary of the extracted data on the content and format of the reviewed questionnaires. This covers the population, domains, categories, items, answer options, anchors, scoring, direction of scale, recall period, administration, questionnaire type and quantification. [file 1477-7525-10-28-S2.DOC]

**Additional file 2: Data extraction results: Content and format of the reviewed questionnaires**

| **Instrument** | **Study** | **Population** | **Number of**  **domains** | **Labelling of**  **domains** | **Category (11 plus ‘other’)** | **No of items** | **Answer options** | **Anchors** | **Scoring** | **Direction of the scale** | **Recall period** | **Administration** | **Questionnaire type** | **Quantification^1)^** |
| --- | --- | --- | --- | --- | --- | --- | --- | --- | --- | --- | --- | --- | --- | --- |
| Questionnaire on Impact of COPD on Activities of Daily Living | Alvarez-Gutierrez 2007 | Patients with COPD | 1 | ADL | Activities of daily living (2) | 7 | 3-point scale | 0=not at all, 2=a lot | Sum | Unidirectional | Unclear | Self-administered | Assessment of the impact of COPD on the ability to perform ADLs (q) | No |
| The Pain Disability Questionnaire (PDQ) | Anagnostis 2004 | Patients with chronic disabling musculoskeletal disorders (CDMDs) | 1 (of 2) | Functional status component (Psychosocial component) | Generic PA (1) | 9 (of 15) | Visual analogue scale | Varies for each question: 0=having no problems at all  1=having the most severe problems you can imagine | A value is scored for each line (every 1.5cm = 1 increment), a sum is scored for the total score and the sub scores | Unidirectional | At this time | Self-administered | Assessment of the impact of pain to perform everyday activities on chronic disabling musculoskeletal patient’s ability (s) | No |
| Chronic Pain Self-efficacy Scale (CPSS) | Anderson 1995 | Chronic pain patients | 1 (of 3) | Self-efficacy for physical function (Self-efficacy for pain management, self-efficacy for coping with symptoms) | PA relating to self (5) | 9 (of 22) | 10-point Likert type scale | 10=very uncertain, 100=very certain | Not reported | Unidirectional | Unclear | Self-administered | Assessment of the self-efficacy to perform various activities (physical function) among chronic pain patients (s) | No |
| The Activities Checklist | Arbuckle 1994 | Elderly | 1 | Work & leisure activities | Work PA (10) | 22 (+ 23^rd^ category “other”) | 5-point Likert scale | 1=less than once a year, 5=daily | Not reported | Unidirectional | No recall period | Self-administered | Assessment of the frequency of the performance of various work and leisure activities in the elderly (q) | No |
| Instrumental Activities of Daily Living (IADL) | Avlund 1993 | Elderly | 2 | IADL-tiredness,  IADL reduced speed | Activities of daily living (2)  Activities of daily living (2) | 14 | Not reported | Not reported | Observed mean score, true IADL score (unclear how they are calculated) | Unidirectional | Unclear | Interviewer administered | Assessment of reduced speed and tiredness (functional ability) in performing IADLs in elderly who are not dependent on help (q) | No |
| Questionnaire of Functional Ability | Avlund 1996 | Elderly | 5 | Mobility-T,  Lower limb-T,  Upper-limb-T,  Mobility H,  PADL-H | PA related mobility (6)  PA related mobility (6)  PA related mobility (6)  PA related mobility (6)  Other (12) | 48 | Categorical | 1=yes, 2=no | Sum of items coded 1 per scale | N/A | Unclear | Unclear | Assessment of the ability (including tiredness and dependency) of elderly people in performing physical activities of daily living (q) | No |
| Chronic Urticaria Quality of Life Questionnaire (CU-Q2oL) | Baiardini 2005 | Patients with Chronic Urticaria | 2 (of 6) | Impact on life activities,  Limits (Pruritus, Swelling, Sleep problems, Looks, Plus additional items not included in final questionnaire) | Generic PA (1)  PA limitation (11) | 9 (of 23) | 5-point Likert scale | 1 = not at all, 5 = very much | Linear transformations of raw scores indicating the percent of maximum possible score were performed. Thus the minimum possible score is defined as 0 and the maximum possible score is defined as 100. | Unidirectional | Unclear | Self-administered | Assessment of the impact of Urticaria on life activities and limits caused by Urticaria (s) | No |
| The Sickness Impact Profile (SIP) | Bergner (1981) | Individuals with a wide range of type and severity of illness | 4 (of 12) | Home management, Mobility, Ambulation, Recreation & pastimes (Body care & movement, Sleep & rest, Eating, Work, Social interaction, Alertness behaviour, Emotional behaviour, Communication) | Household PA (8)  PA related mobility (6)  PA related mobility (6)  Leisure PA (7) | 40 (of 136)  Plus some items from “body care & movement domain” which are in the item pool only | Tick if the statement applies | N/A | Sum of individual domains | N/A | Today | Interviewer administered or self-administered | Assessment of various limitations in body care, home management and movement experienced by patients with a range of illnesses. | No |
| Adapted Falls Efficacy Scale (FES) | Bula 2008 | Elderly receiving postacute, inpatient rehabilitation | 1 | Falls efficacy | PA relating to self (5) | 12 | 11-point Likert-type scale | 0 = none, 10 = full | Sum | Unidirectional | Unclear | Interviewer administered | Assessment of self-efficacy for performing various ADLs without falling in elderly rehabilitation patients (q) | No |
| Fibromyalgia Impact Questionnaire (FIQ) | Burckhardt 1991 | Women with fibromyalgia | 1 (of 5) | Physical function (Anxiety, Depression, Days of work missed, other (not specified)) | Generic PA (1) | 10 (of 19) | 4-point scale | 0 = always able to do,  3 = never able to do | Not reported (can be obtained from author on request) but presumably sum | Unidirectional | During the past week | Self-administered | Assessment of the ability to perform various activities (physical function) in women with fibromyalgia (s) | No |
| Bath Ankylosing Spondylitis Functional Index | Calin 1994 | Patients with ankylosing spondylitis | 1 | Activities of daily living | Activities of daily living (2) | 10 | Visual analogue scale | Easy -  Impossible | 10cm scale divided into 10 to give a score of 0-10. Mean calculated across scales. | Unidirectional | During the last week | Self-administered | Assessment of the level of ability to perform activities of daily living in patients with ankylosing spondylitis (q) | No |
| The Impact on Participation and Autonomy (IPA) | Cardol 1999 | Patients with various disabling conditions | 3 (of 4) | Autonomy in self-care,  Family role,  Mobility & leisure (Social relationship) | PA relating to self (5)  Social PA (9)  PA related mobility (6) | 16 (23) | 5-point Likert scale | 1 = Excellent,  5 = Very poor | Sum of the item scores | Unidirectional | Unclear | Self-administered | Assessment of the possibility of performing various activities in the way they want or when they want in patients with various disabling conditions (s) | No |
| The Impact on Participation and Autonomy Questionnaire (IPAQ) | Cardol 2001 | Patients with chronic illness | 4 (of 5) | Autonomy indoors,  Family role,  Autonomy outdoors,  Paid work & education (Social relations) | PA relating to self (5)  Social PA (9)  PA relating to self (5)  Work PA (10) | 25 (of 39) | 5-point Likert scale | 1 = very good, 5 = very poor | For each domain the participation score and problem-experience score are calculated by summing the item scores. | Unidirectional | Unclear | Self-administered | Assessment of the likelihood of being able to perform various activities in the way they want or when they want in patients with various disabling conditions (s) | No |
| The “Maugeri” Foundation” Respiratory Failure item set (MRF-28), as subdivided by principal components analysis | Carone 1999 | Patients with chronic respiratory failure (CRF) | 1 (of 3) | Daily activity (Cognitive function, Invalidity) | Generic PA (1) | 11 (of 28) | Categorical | True/false/N/A | Positive response = 1  Negative Response = 0  Sum of the scores, expressed as a percentage of maximum score | N/A | No recall period | Not reported | Assessment of the level of ability to perform ADL in patients with respiratory failure (s) | No |
| Zutphen Physical Activity Questionnaire | Caspersen 1991 | Elderly men | 1 | Physical activity | Generic PA (1) | 17 | 4 different types of scale:  Categorical,  3-point scales, 5-point Likert scales,  number of hours spent for an activity | Categorical:  Variants of Yes/no, selecting birds  3-point-scales:  calm-firm, calm-fast  Likert: far more active-far less active, much faster-a lot slower  number of hours:  N/A | Hours a day multiplied with an intensity code. The summary kcal score is in units of kcal/kg of body mass/day | 3-point scales: unidirectional  Likert: unidirectional  Others: N/A | Various: daily, last week, last month, monthly, past 10 years | Not reported | Assessment of ability, frequency and time spent performing daily activities in the elderly (q) | Yes |
| Hong Kong Chinese Everyday Competence Scale | Chou 2003 | Elderly | 6 | Shopping,  Transportation,  Symbols used in daily life,  Utilisation of common electric appliances,  Visiting doctors,  Money management | Leisure PA (7)  PA related mobility (6)  Other (12)  Household PA (8)  Other (12)  Other (12) | 36 | Categorical | 0, 1 = correct answer | Sum (ranging from 0 to 36) | N/A | Unclear | Interviewer administered | Unknown - questionnaire unavailable. | Unknown |
| The Adelaide Activities Profile (AAP) | Clark 1995 | Elderly | 4 | Domestic chores,  Household maintenance,  Service to others,  Social activities | Household PA (8)  Household PA (8)  Social PA (9)  Social PA (9) | 21 | 4-point scale | Different labelling per question, pattern:  0 = Never, 3 = most frequently (specified per question) | Not reported | N/A | Typical 3 month period | Interviewer administered | Assessment of the frequency of which various life-style activities are performed in a typical three-month period by the elderly (q) | No |
| Steadiness Score | Clark 2005 | Elderly | 1 | Steadiness | PA related mobility (6) | 3 | 4-point scale | Lowest number = very steady, highest number = very unsteady | Sum | Unidirectional | At this time | Self-administered | Assessment of the level of steadiness when performing three basic activities of daily living in the elderly (q) | No |
| The Customary Activity Questionnaire | Dallosso 1988 | Elderly | 4 | Outdoor productive activities, Indoor productive activities, Leisure activities,  Walking | Household PA (8)  Household PA (8)  Leisure PA (7)  PA related mobility (6) | 4 | Outdoor, indoor and leisure activities:  1) categorical  2) If yes, frequency and duration of the activity in minutes per week  walking: duration on a typical day | Outdoor, indoor, leisure activities:  1) yes/no  2) N/A  Walking:  N/A | Not reported | N/A | Outdoor, indoor leisure activities: not reported  Walking: yesterday, (if atypical an earlier day, up to a maximum of 6 days previously. Using a recall method, the interviewer went through the day in blocks of time) | Interviewer-administered | Unknown - questionnaire unavailable. | Uknown |
| Exercise Self-Regulatory Efficacy Scale (Ex-SRES) | Davis 2007 | COPD | 1 | Exercise self-regulatory efficacy | PA relating to self (5) | 16 | Numerical scale % | 0 =not at all confident  100 = highly confident | Sum | Unidirectional | No recall period | Self-administered | Assessment of the confidence in continuing to exercise regularly when faced with various impediments in COPD patients (q) | No |
| The YALE Physical Activity Survey for Older Adults (YPAS) | Dipietro 1993 | Elderly | 12 | Work,  Yard work, Caretaking, Exercise, Recreational activities,  Vigorous activity, Leisure walking, Moving, Standing,  Sitting,  Flight,  Seasonal adjustment | Work PA (10)  Household PA (8)  Social PA (9)  Exercise PA (4)  Leisure PA (7)  Exercise PA (4)  Leisure PA (7)  PA related mobility (6)  PA related mobility (6)  PA related mobility (6)  PA related mobility (6)  Other (12) | 43 | 6 different types of scale: no. of hours per week,  5-point Likert scales with 1 or 2 N/A options,  4-point scales with 2 N/A options,  3-point scales with 3 N/A options,  6-point Likert scales with 2 N/A options | One part: N/A  Other parts: various | Activity checklist:  1) Total time: summing the time for each activity, expressed in [h per week]  2) Energy expenditure: time * intensity code, then summed  3) Activity dimensions: frequency score*duration score*weighting factor (for each of 5 specific activities)  Activity dimensions:  Frequency score * duration * weight | N/A | Various, mostly during the past month | Interviewer administered | Assessment of total time spent doing various physical activities and frequency and duration of specific activities in the elderly (q) | Yes |
| Adapted Barthel Index | Dorevitch 1992 | Elderly | 1 | No labelling | Other (12) | 13 | 3-point scale | 0 = capable to perform alone  1 = capable to perform with assistance  2 = can't perform even with assistance | Weighting based on the experience and clinical judgement of the original authors  A summary score can be calculated  (unsure, S. 793, upper left edge) | Unidirectional | No recall period | Self-administered | Assessment of the capacity to perform activities of daily living with or without assistance in the elderly (q) | No |
| Chronic Heart Failure Assessment Tool (CHAT) | Dunderdale 2008 | Heart Failure patients | 2 (of 4) | Activity levels,  Symptoms (psychological aspects, emotions) | Generic PA (1)  Dyspnoea and Symptom related PA (3) | 23 (of 40) | 5-point Likert scales  4-point scale | 5-point: 0=Much less than other adults to 4=much more than other adults, 0=never to 4=very often  4-point: 0=not limited at all-3=always limited | Sum of domain | Unidirectional | Past two weeks | Self-administered | Assessment of limitations and symptoms when performing ADLs in heart failure patients, in comparison to healthy adults of the same age (s) | No |
| University of California San Diego Shortness of Breath Questionnaire (SOBQ) | Eakin 1998 | Chronic lung disease | 1 | Shortness of breath | Dyspnoea and Symptom related PA (3) | 24 | 6-point Likert type scale | 0 = not at all  5 = maximal or unable to do because of breathlessness | Sum | Unidirectional | During the past week | Self-administered | Assessment of the level of shortness of breath/dyspnoea while performing ADLs in chronic lung disease patients (q) | No |
| Meaningful Activity Participation Assessment (MAPA) | Eakman 2007 | Elderly | 5 | A) MAPA FREQUENCY  B) MAPA MEANING  C) MAPA HEALTH  D) MAPA REASONS  E) MAPA GLOBAL | Other (12)  Other (12)  Other (12)  Other (12)  Other (12) | A) 28  B) 28  C) 28  D) 28  E) 8 | A) 7-point-Likert type scale B) 5 point Likert type scale  C) 5 point Likert-type scale  D) Categorical 6 categories  E) 7 point Likert scale | A) 1= not at all/ 7 = everyday  B) 1 = not at all meaningful, 5 = extremely meaningful  C) 1 =Extremely unhealthy/ 5 = Extremely Healthy  D) 1 = Need or Have to do this, 2 = for new experiences, 3 = connect with people, 4 = sense of accomplishment, 5 = other, 6 = I don't do this  E) 1= not at all/ 7= a great deal: item 1-7, 1= a great deal less; 7= a great deal more: item 8 | Sum of all scales, Count of activities raised in A = Diversity, B/Diversity, C/ Diversity, AxB and AxC, | A) 1=not at all, 2=every day  B) unidirectional  C) Bi directional ?  D) not applicable  E) 1-7 unidirectional, 8 = bidirectional | A) last few months  B) no recall period  C) no recall period  D) no recall period  E) no recall period | Self-administered | Assessment of the meaningfulness and frequency of various activities among the elderly (q) | No |
| Part A of the ORAS Multidimensional Functional Assessment Questionnaire (OMFAQ) | Fillenbaum 1981 | Different populations of older adults as well as for “specific individuals” | 1 (of 5) | Self-care capacity  (Physical health  Social, Economic, Mental health) | PA relating to self (5) | 22 (of 65) | Categorical,  binary,  3-point scale,  4-point scale | Various (to many to list) | Sum  “A final summary rating was developed for each dimension, with possible scores ranging from excellent functioning to totally impaired” | Unidirectional | No recall period | Interviewer-administered | Assessment of the ability to perform ADLs with or without any help and of the use of aids in general in various elderly populations (s) | No |
| The Five Item Instrumental Activities of Daily Living Scale | Fillenbaum 1985 | Elderly | 1 | N/A | Other (12) | 5 | Categorical | 1=without help  2=with help | Guttman scale | N/A | No recall period | Self-administered | Assessment of the ability to perform IADL with or without help in the elderly (q) | No |
| The magnitude estimation scale | Finch 1995 | Elderly people / patients at long-term-care | 2 | ADL,  IADL | Activities of daily living (2)  Activities of daily living (2) | 13 | 2, 3 and 4-point scales | Varies by question e.g. needs complete assistance – needs little assistance | Sum of the weighted items, separately calculated for the 2 domains | Unidirectional | Unclear - Questionnaire not available | Not reported | Assessment of the degree of dependence in performing ADL and IADL in the elderly or patients at long-term-care (q) | No |
| Daily Activity Diary for Chronic Pain Patients | Follick 1984 | Chronic pain patients | 2 (of 6) | Position,  Major activity (Medications, Pain Relief Activities or Devices, Daily Pain Rating, Daily Rating of Mood and Tension) | PA related mobility (6)  Generic PA (1) | 9 (of 13) | Diary | N/A | Mean score | N/A | 3 times a day, during the past week | Self-administered | Assessment of the time spent in daily activities and postures in chronic pain patients via a diary (s) | Yes |
| Household Activities - Activities of Daily Living Scale (HHA-ADL) | Frederiks 1990 | Elderly people living at home | 2 | Household activities  Activities of daily living | Household PA (8)  Activities of daily living (2) | 13 | Categorical | Yes/no | Not applicable (Guttman scale) | Not applicable (categorical) | No recall period/ unclear (presumably at that moment) | Self-administered | Assessment of the ability to undertake various household activities and ADLs in elderly people (q) | No |
| The Lifetime Total Physical Activity Questionnaire | Friedenreich 1998 | Middle aged and elderly women | 3 | Lifetime occupational activities,  Lifetime household activities,  Lifetime exercise/sports activities | Work PA (10)  Household PA (8)  Exercise PA (4) | N/A (depends on no. of activities the respondent does) | Duration, frequency and intensity for each activity  Intensity is used for occupational activities only and is assessed by a 4-point-scale | Duration and frequency: N/A  Intensity: 1=sedentary  2=light  3=moderate  4=heavy activities | Total physical activity was estimated as the sum of occupational, household and exercise/sports activities in hours per week. It is also possible to convert these data into energy expended by multiplying the hours spent by the estimated metabolic cost of that activity. The resulting data would be donated as MET-hours/week (MET=ratio: associated metabolic rate for a specific activity/the resting metabolic rate | Duration and frequency: N/A  Intensity: Unidirectional | Over/during the lifetime or At least 8 hours a week for four months of the year over your lifetime | Interviewer-administered (needs interviewers trained in cognitive interviewing methods) | Assessment of lifetime patterns of total physical activity including the duration, frequency and intensity of occupational, household and exercise//sports activities (q) | Yes |
| Health Assessment Questionnaire | Fries 1982 | Rheumatoid arthritis patients | 1 (of 3) | Disability (Discomfort & pain,  Global health assessment) | PA limitation (11) | 20 (of 22) | 4-point scale | 0 = without any difficulty, 3 = Unable to do | Sum | Unidirectional | Over the past week | Interviewer administered, self-administered or telephone administered | Assessment of the ability to carry out daily activities in arthritis patients (s) | No |
| Population surveys of chronic disease and disability (Section I) | Garrad 1971 | Disabled persons | 3 (of 4) | Mobility,  Self-care,  Domestic duties (Occupation) | PA related mobility (6)  PA relating to self (5)  Household PA (8) | 18 (of 19) | Inconsistent:  1. combined: categorical, followed by x-point scale  2. categorical only  3. x-point scale only | Inconsistent | Not reported | N/A | No recall period | Interviewer-administered | Assessment of ability to perform different ADLs and mobility activities in disabled persons (s) | No |
| London Chest Activity of Daily Living Scale (LCADL) | Garrod 2000 | Patients with severe COPD | 4 | Self-care activities,  Domestic activities,  Physical activities,  Leisure activities | PA relating to self (5)  Household PA (8)  Generic PA (1)  Leisure PA (7) | 15 | 5-point Likert scale | (0=Wouldn't do anyway)  1=I do not get breathless  5= Someone else does this for me | Total score | Unidirectional | The last few days | Self-administered | Assessment of the level of breathlessness during various domestic, self-care, physical and leisure activities in COPD patients (q) | No |
| The Chronic Respiratory Disease Questionnaire (CRQ) | Guyatt 1987 | patients with chronic airflow limitations | 1 (of 4) | Feeling short of breath (fatigue, emotional function, mastery = feeling of control over the disease and its effects) | Dyspnoea and Symptom related PA (3) | 5 (of 20)  (patients elicits 5 activities in which he experiences dyspnoea, 26 activities are offered as suggestions to aid recall) | 7-point Likert type scale | 1=extremely short of breath  7=not at all short of breath | Mean scores for all four dimensions | Unidirectional | Last 2 weeks | Interviewer-administered | Assessment of the level of shortness of breath while doing the 5 most important activities in patients with chronic airflow limitations (s) | No |
| The Geriatric Quality of Life Questionnaire (GQLQ) | Guyatt 1993 | Frail elderly | 1 (of 3) | ADL (Symptoms, emotional functions) | Activities of daily living (2) | 8 (ADL domain) + 1 (symptom domain) (of 25)  additional info:  patients are asked to chose 8 ADLs / 8 symptoms, each out of a list containing 24 items | Categorical  7-point Likert type scale | If respondents had needs of help:  1=great deal of help  7=almost no help at all  if respond ants reported, that they didn't need help, but had difficulty with the activity, the following difficulty graduation were used:  1=a very great deal of difficulty  7=almost no difficulty | “Questionnaire domain scores were calculated by adding up the importance ratings for each item in a particular domain labelled by a subject as a problem to produce a total score for each domain for each person.” | Unidirectional | Past 2 weeks | Interviewer-administered | Assessment of the graduation of needed help or difficulty in performing activities in frail elderly (q) | No |
| London Handicap Scale | Harwood 1994 | Patients with various chronic conditions | 3 (of 6) | Getting around,  Looking after yourself,  Work & leisure (Getting on with people, Awareness of your surroundings, Affording things you need) | PA related mobility (6)  PA relating to self (5)  Work PA (10) | 3 (of 6) | 6-point Likert type scale | 1 = not at all,  6 = completely | Weighted sum | Unidirectional | Over the last week | Self-administered | Assessment of the impact of health on patients’ everyday lives (s) | No |
| Osteoporosis Functional Disability Questionnaire | Helmes 1995 | Patients with osteoporosis | 1 (of 5) | Activities of daily living (General health & extent of pain,  Depression, Social & recreational activities, Program benefits) | Activities of daily living (2) | 26 (of 59) | 5-point Likert scale | 0 = absolutely unable to do,  4 = able to do without effort | Sum | Unidirectional | Not reported | Self-administered | Assessment of ability to perform day-to-day activities in patients with osteoporosis (s) | No |
| The Duke Activity Status Index (DASI) | Hlatky 1989 | Congestive heart failure (CHF) or cardiovascular disease | 1 | N/A | Other (12) | 12 | 1) Categorical  2) Categorical | 1) Yes/no  2) If yes, easy/hard | Weighted items (MET) | N/A | No recall period | Self-administered | Assessment of the ability to perform and difficultly performing various activities (q) | No |
| Multiple Sclerosis Walking Scale (MSWS-12) | Hobart 2003 | Patients with multiple sclerosis | 1 | Walking ability | PA related mobility (6) | 12 | 5-point Likert scale | 1 = not at all,  5 = extremely | Total score, transformed to a scale with a range from 0 to 100 | Unidirectional | Past two weeks | Self-administered | Assessment of limitations in the walking ability due to MS (q) | No |
| Activities Index | Holbrook 1983 | Stroke patients | 3 | Domestic chores,  Leisure/work activities,  Outdoor activities | Household PA (8)  Leisure PA (7)  Household PA (8) | 15 | 4-point scales | 1 = never to 4 = most days,  1 = never to 4 = at least weekly,  1 = none to 4 = all necessary,  1 = none to 4 = over 1 a fortnight,  1 = none to 4 = over 30 h/week | Not reported | Unidirectional | For day to day activities: over the past 3 month  For more seasonal items: over the past 6 month | Not reported, presumably interviewer administered | Assessment of the frequency of performance of domestic, leisure/work and outdoor activities in stroke patients (q) | No |
| The Living with Asthma Questionnaire | Hyland 1991 | Individuals with asthma | 2 (of 11) | Work & other activities,  Mobility (Social / leisure, sport, holidays, sleep, colds, medication usage, effects on others, doctors, dysphoric states and attitudes) | Work PA (10)  PA related mobility (6) | 12 (of 68) | 3 point scale with N/A option | Very true of me, Slightly true of me,  Untrue of me + a not applicable option | Positive items: very true = 0, untrue = 2.  Negative items: very true = 2, untrue = 0. N/A always = 0.  Sum of the scores for all 68 questions divided by the number of questions which have either had a very true, slightly true or untrue response | Bi-directional | No recall period | Self-administered or interviewer administered | Assessment of the subjective experience in specific physical activity domains (mobility, work & other activities) in asthma patients (s) | No |
| Respiratory Illness Questionnaire-monitoring 10 (RIQ-MON10) | Jacobs 2004 | Adults with chronic respiratory diseases in routine primary care | 1 (of 2) | Experienced physical & social limitations (physical and emotional complaints) | PA limitation (11) | 5 (of 10) | 7-point Likert type scale | 1=not troubled at all  7=cannot do so, too much troubled by the disease | Mean score for each subscale and the whole scale | Unidirectional | During the past 4 weeks | Self-administered | Assessment of the level of being troubled because of breathing problems in performing activities (s) | No |
| Functional Status Assessment Instrument | Jette 1978 | Arthritis | 3 | Mobility,  Self-care,  Work | PA related mobility (6)  PA relating to self (5)  Work PA (10) | 88 (of 132) (items rating Degree of dependence (44)/ Difficulty (44) / pain (44)) | Difficulty:  4 point scale  Degree of dependence:  5-point-Likert-type scale | 0 = independent,  1 = uses mechanical equipment,  2 = uses human assistance,  3 = uses both,  4 = can not perform activity  1 = no difficulty;  2 = mild difficulty  3 = moderate difficulty  4 = severe difficulty | Sum | Unidirectional | No recall period | Interviewer administered or self-administered | Assessment of the degree of dependence in the usual performance of ADL in adults with chronic respiratory diseases (q) | No |
| The St. George's Respiratory Questionnaire (SGRQ) | Jones 1992 | People with chronic airflow limitations | 1 (of 3) | Activity (symptoms, impacts (on daily life) | Generic PA (1) | 25 (of 50, 76 weighted responses) | Categorical | categorical:  true/false (for all PA domains) | “Calculated from the summed weights for the positive responses” (taken from copy of questionnaire). | Likert-type-Scale-questions:  Unidirectional  categorical: N/A | "These days" / "usually" | Self-administered | Assessment of limitations due to breathlessness in performing physical activities in people with chronic airflow limitations (s) | No |
| The Quality of Well-Being Scale, Version 1.04 (QWB) | Kaplan 1997 | Wide variety of population (not further specified) | 3 (of 5) | Self-care, Physical Activity  Usual Activity  (Acute and chronic symptoms, Mobility) | PA relating to self (5)  Generic PA (1)  Generic PA (1) | 14 (of 71) | Categorical  Free report of activities  11-point scale | Categorical (more than one answer possible): a=No days  b=Yesterday  c=2 days ago  d=3 days ago  Free report of activities: N/A  11-point scale:  0=the least desirable state of health that you could imagine  100=perfect health | Total score  (Weighted score for symptom complex, added to each of the other [unweighted] scores)  The final score reflects well-being from 0 to 1.000 | Unidirectional | Last three days | Self-administered (but a Quality of Life Assessor must be available at visits to answer questions and review completed forms) | Assessment of the frequency of limitations experienced by patients during the last 3 days (s) | No |
| Functional Independence Measure (FIM) | Keith 1987 | Patients at rehabilitation stage | 3 (of 6) | Self-care, Transfers, Locomotion (Sphincter control, Communication, Social & cognitive skills) | PA relating to self (5)  PA related mobility (6)  PA related mobility (6) | 12 (of 18) | 7-point Likert type scale | 1= total assistance,  7 = complete independence | Total score | Unidirectional | No recall period | Interviewer administered | Assessment of the amount of assistance needed in performing activities of daily life in rehabilitation patients (s) | No |
| Hierarchical Polychotomous ADL-IADL Scale (developed further to Groningen Activity Restriction Scale (GARS) (Kempen et al., 1996) | Kempen 1990 | Non-institutionalised elders | 2 | ADL,  IADL | Activities of daily living (2)  Activities of daily living (2) | 18 | 3-point scale | 1=perform the activity independently and easily  2=independently but with some difficulty  3=dependent on others or supervised for the execution of a certain activity | Guttman scale, Mokken scale | Unidirectional | Not reported | Interviewer-administered | Assessment of the degree of independence in performing ADLs and IADLs in the elderly (q) | No |
| COPD-Disability-Index (CDI) | Kühl 2009 | Patients with COPD | 1 | COPD disability index | PA limitation (11) | 7 | 11-point Likert type scale | 0=no limitations  10=fully limited | Total score (sum) | Unidirectional | No recall period | Self-administered  interviewer-administered | Assessment of limitations in different areas of life due to breathing problems in COPD patients (q) | No |
| The Pulmonary Functional Status and Dyspnoea Questionnaire (PFSDQ) | Lareau 1994 | Patients with COPD | 2 domains, 6 activity subscales | Domains: dyspnoea intensity with activities and functional ability  Subscales: self-care  mobility  eating  home management  social  recreational | Dyspnoea and Symptom related PA (3)  Generic PA (1) | X (of 164) | Dyspnoea component:  11-point Likert type scale  functional ability component:  7-point Likert type scale  categorical (3 options [Question No. 2]) | Dyspnoea component:  0=none  10=very severe  functional ability component:  1=as active as I've ever been  7=have omitted entirely  categorical:  "experienced severe to very severe dyspnoea"  daily / weekly or monthly | Dyspnoea component:  sum of dyspnoea score divided by the number of rated activities  functional ability component:  sum of functional ability score divided by the number of rated activities | Unidirectional | At the present time | Self-administered | Assessment of the impact of dyspnoea on ability to perform daily activities in COPD patients (q) | No |
| The modified version of the Pulmonary Functional Status and Dyspnoea Questionnaire (PFSDQ-M) | Lareau 1998 | Patients with pulmonary impairment | 3 | Activity,  Dyspnoea,  Fatigue | Generic PA (1)  Dyspnoea and Symptom related PA (3)  Dyspnoea and Symptom related PA (3) | 40 | 11-point Likert type scale | Activity, Dyspnoea, Fatigue domain:  0=as active as I've ever been  10=omitted the activity entirely  Additionally for Dyspnoea and Fatigue domain:  0=no shortness of breath/tiredness  10=Very severe shortness of breath/tiredness | Dyspnoea with activity (DA) and change experienced by the patient with activities (CA) are designed to be used as separate summated scores, whereas the general dyspnoea (GA) subscale is designed to be descriptive and not a combined score. | Unidirectional | Unclear | Self-administered | Assessment of current activity levels compared to past self in the performance of various daily activities, and an assessment of the intensity of dyspnoea and fatigue with these activities in patients with pulmonary impairment (q) | No |
| University of Cincinnati Dyspnoea Questionnaire (UCDQ) | Lee 1998 | Patients with pulmonary diseases | 3 | Breathlessness during physical activity,  Breathlessness during speaking activity,  Breathlessness when speaking during physical activity | Dyspnoea and Symptom related PA (3)  Dyspnoea and Symptom related PA (3)  Dyspnoea and Symptom related PA (3) | 30 | 5-point-Likert type scale + option to mark “not interested” | 1=Not at all shortness of breath (SOB)  3=Occasionally SOB  5=Always SOB or cannot do | Not reported | Unidirectional | No recall period | Self-administered &  interviewer- administered | Assessment of the level of shortness of breath during physical activities, speech activities, and combinations of the 2 in patients with pulmonary diseases (q) | No |
| The Functional Performance Inventory (FPI) | Leidy 1999 | Patients with COPD | 6 | Body care,  Maintaining the household,  Physical exercise,  Recreation activities for personal pleasure,  Spiritual activities,  Social interaction -family & friends | PA relating to self (5)  Household PA (8)  Exercise PA (4)  Leisure PA (7)  Other (12)  Social PA (9) | 67 | 4-point scale with N/A option | 1, 2, 3=do with: no, some, much difficulty  4=don't do because Health Reasons  N/A=don't do because choose not to | Mean of all items (potential range from 0 to 3) | Unidirectional | No recall period | Self-administered | Assessment of the degree of difficulty in performing day-to-day-activities in COPD patients (q) | No |
| Work Limitations Questionnaire (WLQ) | Lerner 2001 | Not clearly described, but presumably workers with chronic health problems | 1 (of 4) | Physical demands (Time management, Mental-interpersonal demands,  Output demands) | Generic PA (1) | 6 (of 25) | 5-point Likert scale plus N/A option | 1 = all of the time,  5 = none of the time  0 = does not apply to my job | Summated average | Unidirectional | Past two weeks | Self-administered | Assessment of the level of ability to perform work activities in patients with chronic condition (s) | No |
| The Asthma Impact Record (AIR) Index | Letrait 1996 | Asthmatic patients | 1 (of 4) | Physical activities (physical symptoms, psychological, social dimensions) | Generic PA (1) | Unknown | Tick if statement applies | N/A | Presumably sum (Negative items: score of 1 if ticked and 0 if not ticked,  Positive items: score of 0 if ticked and 1 if not ticked) | N/A | Not reported | Self-administered | Unknown - questionnaire unavailable. | Unknown |
| The Cardiovascular Limitations and Symptoms Profile (CLASP) | Lewin 2002 | Patients with angina and angiographically established CHD | Domains: 1 (of 2)  Subscales within domain: 3 (of 5) | Domains: Functional Limitations (Symptoms)  Subscales within functional limitations domain:, mobility, social life and leisure activities, activities within the home (concerns and worries, gender) | PA related mobility (6)  Social PA (9)  Household PA (8) | 11 (of 37) | 4-point scales,  5 & 7 point Likert type scales | Various (too many to list) | Scores are weighted to provide a total for each subscale, categorizing symptoms as mild, moderate, or severe and limitations as normal (no limitation), mild, moderate, or severe. | Uni-directional | Over the last 2 weeks | Self-administered | Assessment of the level of ability to perform activities and the limitation in performing activities in patients with angina and angiographically established CHD (s) | No |
| Activities of Daily Living Scale (ADL-scale) | Linton 1990 | Patients with chronic pain | 1 | Activities of daily living | Activities of daily living (2) | 10 | 11-point Likert scale | Not reported | Not reported | Unidirectional | Unclear | Self-administered | Assessment of impairment in performing activities of daily living in chronic pain patients (q) | No |
| The Physical Activity Questionnaire | Liu 2001 | Healthy adults, adults with diseases, risk groups such as the elderly | 5 | Occupation,  General activity,  Care taking,  Exercise,  Leisure activities | Work PA (10)  Generic PA (1)  Social PA (9)  Exercise PA (4)  Leisure PA (7) | 55 | Indication of intensity:  Hour's spent for each time (_h)  Frequency per week (_times) | Not relevant | Calculation of the total daily energy expenditure (TEE). Sum of: energy intensity code (kcal/min) of each activity * time interval (min) and frequency | Unidirectional | During the last week  If the last week does not represent a typical week, a usual week as reference is asked | Self-administered | Assessment of participation in common occupational and leisure time activities and the time spent for those activities (q) | Yes |
| The Quality of Life Respiratory Illness Questionnaire (QOL-RIQ) | Maillé 1997 | Patients with mild to moderate chronic non-specific lung disease | 3 (of 7) | Problems with general activities,  Daily domestic activities,  Social activities relationships & sexuality (Breathing problems, Physical problems related to chest problems, emotions related to chest problems, Situations that might trigger or enhance breathing problems) | PA limitation (11)  Household PA (8)  Social PA (9) | 21 (of 55) | 7-point Likert type scale | 1=Not at all  7=Extremely | Total score (Not exactly reported) | Unidirectional | During the 12 month prior to the investigation | Self-administered | Assessment of the degree of being troubled with general activities, and the degree of being troubled with breathing problems with daily domestic activities and social activities, relationships and sexuality in lung disease patients (s) | No |
| The Leisure Time Physical Activity Instrument (LTPAI) | Mannerkorpi 2005 | Patients with rheumatoid diseases, patients with long-lasting pain | 1 | Average activity level | Generic PA (1) | 4 | For the items light, moderate and severe exercise the spent time in hours a week is asked, using a 3-point scale | 3-point scale:  1=0.5-1.5h  2=2-4h  3=>4h | The mean value of the first two steps, being 1 and 3 hours, were used in the calculation of the total score. If no step was selected, the number of hours for the category was 0. The hours of the intensity categories were added together to produce the Leisure Time Physical Activity during a week. | Unidirectional | Over the past month | Self-administered | Assessment of the amount of time (in categories) spent on 4 different intensity levels of activity in rheumatoid disease and long-lasting pain patients (q) | No |
| The Physical Activity at Home or at Work Instrument (PAHWI) | Mannerkorpi 2005 | Patients with rheumatoid diseases  Patients with long-lasting pain | 2 | Work at home,  Workplace | Household PA (8)  Work PA (10) | 7 | No. of hours per week | No anchors | The hours are added  together to produce the total score for the  PHWAI, activity at home and activity at workplace. | N/A | During the space of a week | Self-administered | Assessment of the time spent for different intensity levels of activities in rheumatoid disease and long-lasting pain patients (q) | Yes |
| The Immune Thrombocytopenic Purpura Patient Assessment Questionnaire (ITP-PAQ) | Mathias 2007 | Adults with immune thrombocytopenic purpura (ITP) | 2 (of 10) | Activity,  Social activity (symptoms, fatigue, bother, psychological, fear, overall quality of life, menstrual symptoms, fertility and work) | Generic PA (1)  Social PA (9) | 6 (of 44) | 5-point Likert scale | Activity and 2 questions of social activity: extremely - not at all  Other 2 questions of social activity: all the time - never | All scores are converted to a 0-100 scale. The scale score is the mean of all items in the scale. Scale Score ranges from 0 to 100, with higher scores indicating better health status | Unidirectional | In the past 4 weeks | Self-administered | Assessment of the limitation in performing physical activities due to ITP in adults with immune thrombocytopenic purpura (s) | No |
| Scale for the Instrumental Activities of Daily Living in the Elderly (IADL-EDR) | Mathuranath 2005 | Elderly people | 5 | Community activities,  Household activities,  Self-care items,  Cognitive activities,  Social/recreational activities | Social PA (9)  Household PA (8)  PA relating to self (5)  Other (12)  Social PA (9) | 11 | 1. Applicability: categorical  2. Degree of disability: 3-point Likert-type scale  3. Underlying impairment: categorical | 1. yes/no  2. 0=able to complete it competently  2=unable to perform  3. Underlying impairment: cognitive/physical/both | Physical disability index: Sum of disability ratings due to physical impairment, divided by the product of the maximum possible "disability" on an item and the number oft items reported applicable  Cognitive disability index (same procedure) | Unidirectional | No recall period | Interviewer-administered | Assessment of the applicability, degree of disability and underlying impairment in performing instrumental activities of daily living in the elderly (q) | No |
| The Multidimensional Task Ability Profile (MTAP) | Mayer 2005 | Musculoskeletal disorders | X of X  Questionnaire not available (Computer program) | Not reported Questionnaire not available (Computer program) |  | X (of 111) Questionnaire not available (Computer program) | 5-point-Likert type scale | 1=Able  5=Unable | Weighted scoring (not specified) | Not reported | Not reported | Self-administered | Unknown - questionnaire unavailable. | Unknown |
| MOS 36-Item Short-Form Health Survey (SF-36) | McHorney 1994 | “General Populations”  “Diverse Populations” | 4 (of 8) | Physical functioning,  Role physical,  Role emotional,  Social functioning (Body pain (BP), general health perceptions (GH), vitality (VT), mental health (MH), reported change (TRAN)) | Generic PA (1)  PA limitation (11)  Other (12)  Social PA (9) | 19 (of 36) | Categorical  3-point scale  5-point Likert scales | Categorical: yes/no  3-point scale:  Yes limited a lot, Yes limited a little, No not limited at all  5-point Likert scales:  not at all - extremely, &  all of the time – none of the time | ‘Likert method of summated ratings’ | 3-point scale & 5-point Likert scales: unidirectional  Categorical: N/A | “During the past for weeks”, “During a typical day”  or no recall period | Self-administered or interviewer administered | Assessment of limitations in performing activities (s) | No |
| The Dyspnoea Management Questionnaire (DMQ) | Migliore 2006 | Adults with COPD and asthma | 2 | Dyspnoea & related anxiety with activities (Appraisal of dyspnoea coping skills) | Dyspnoea and Symptom related PA (3)  Dyspnoea and Symptom related PA (3) | 30 | 7-point Likert-type scale | Inconsistent  ranging from 0 to 6, a higher score represents a better functional status | Sum of score divided by number of items for each subscale | Unidirectional | No recall period | Self-administered | Assessment of the level of shortness of breath while performing activities and appraisal of dyspnea coping skills in COPD and asthma patients (q) | No |
| The Centres for Disease Control and Prevention’s Healthy Days Measures (the CDC HRQOL-14) | Moriarty 2003  Atlanta Georgia CDC 2000 [http://www.cdc.gov/hrqol/] | “several populations” | 1 (of 3) | Activity Limitations Module  (Healthy Days Core Module, Healthy Days Symptoms Module) | PA limitation (11) | 5 (of 14) | Two different categorical scales  4-point-scale  Plus N/A options for each question | Categorical:  Yes/no & option to chose one of several diseases/problems  4-point scale: 1=Days, 2=Weeks, 3=Months, 4=Years  N/A options: Don’t know/not sure or  Refused | Summary score: Sum of unhealthy days in the past 30 days (1 unhealthy day is counted regardless whether mental, physical health or both of them are reported by patient in a day) | Categorical: N/A  4-point-Likert-type scale: Unidirectional | During the previous 30 days | Interviewer-administered | Assessment of the limitation in performing daily life activities because of the impairment or health problem (s) | No |
| The Chronic Obstructive Pulmonary Disease Activity Rating Scale (CARS) | Morimoto | Patients with COPD | 4 | Self-care activity,  Domestic activity,  Outdoor activity,  Social interaction activity | PA relating to self (5)  Household PA (8)  PA related mobility (6)  Social PA (9) | 12 | 3-point scale | 0=dependent  1=partially dependent  2=completely independent | Total score and sub scores on domains | Unidirectional | Not reported | Self-administered | Assessment of the ability to perform life-related activities independently in COPD patients (q) | No |
| The IOWA Self-Assessment Inventory (ISAI | Morris 1989 | Elderly persons | 1 (of 6) | Activities of daily living (Social resources, economic resources, mental health, physical health, cognitive status) | Activities of daily living (2) | 20 (of 120) | 4-point scale | True,  More often true than not,  More often false than not,  False | Not reported | Not reported | Not reported | Self-administered | Unknown - questionnaire unavailable. | Unknown |
| The Veterans Specific Activity Questionnaire (VSAQ) | Myers 1994 | Veterans | 1 | Physical activity | Generic PA (1) | 13 | Mark if it applies | N/A | Guttman scale | N/A | No recall period | Self-administered | Assessment of the degree of an activity-intensity (in METs), that causes the patient to want to stop the activity in veterans (q) | No |
| The Chronic Fatigue Syndrome-Activities and Participation Questionnaire (CFS-APQ) | Nijs 2003 | Patients with chronic fatigue syndrome (CFS) | 1 | Activities & participation | Generic PA (1) | 26 | 4-point scale plus N/A option | satisfaction section:  1=totally no agreement, 4=totally agree  importance verification:  1=This activity is not at all important, 4=very important | 2 approaches:  Total score 1: sum of the single-item scores (satisfaction-score multiplied by the score on the importance verification) divided by the number of completed questions  Total score 2: sum of satisfaction score divided by number of properly completed question  5 items measure participation 21 activity | Unidirectional | During the past 7 days | Self-administered | Assessment of limitations and the degree of importance of these limitations in performing normal daily activities due to CFS symptoms (q) | No |
| Daily Living Activities Questionnaire | Nouri, 1987 | Stroke patients | 4 | Mobility,  In the kitchen,  Domestic tasks,  Leisure activities | PA related mobility (6)  Household PA (8)  Household PA (8)  Leisure PA (7) | 22 | 4-point scale | 0 = with help, no  1 = on my own, on my own with difficulty | Guttmann scaling | Unidirectional | No recall period | Self-administered (can also be interviewer administered) | Assessment of the ability to perform activities of daily living with or without help in stroke patients (q) | No |
| The Duke-UNC Health Profile | Parkerson, 1981 | Patients with various chronic conditions | 2 (of 4) | Physical function, Social function (Symptom status, Emotional function) | Generic PA (1)  Social PA (9) | 14 (of 63) | 3-point scales | Physical function: 2 = none, 0 = 5-7 days and 2 = none, 0 = a lot  Social function: 5-7 days – not at all | A separate score is calculated for each domain by dividing the individual’s score for that domain by the maximum possible score for that domain. This results in a proportion ranging from 0 for the worst possible to 1 for the best possible health status. | Unidirectional | Past week and Today | Self-administered or interviewer administered | Assessment of the frequency and difficulty in performing activities in chronic patients (s) | No |
| The harmonized four-item ADL measure | Pluijm 2005 | older people | 1 | No labelling |  | 4 | Categorical | Not applicable (1 = need help 0 = not need help) | Total score & categorisation from total score > 0 = 1 | Not applicable | Unclear | Interviewer-administered | Assessment of dependence in performing ADLs in older people (q) | No |
| Specific Activity Questionnaire (SAQ) | Rankin 1996 | Cardiac patients | 1 | Physical activity | Generic PA (1) | 13 | Categorical | Yes/no | Recording the corresponding MET value of the most demanding activity that the subject perceived he or she could complete without symptoms | N/A | No recall period | Self-administered | Assessment of the ability to perform activities without symptoms, by measuring the degree of an activity-intensity (in METs), which cardiac patients are able to complete without symptoms (q) | No |
| The Peripheral Arterial Disease (PAD) Walking Impairment Questionnaire | Regensteiner 1990 | Patients with peripheral arterial disease (PAD) | 2 (of 3) | Walking distance,  Walking speed (Walking impairment) | PA related mobility (6)  PA related mobility (6) | 10 (18) | 4-point scale | 0=did not do  3=no difficulty | Summary score divided by maximal possible summary score  Summary score for  1) Walking distance:  Degree of difficulty multiplied with score weight (=distance in feet)  2) Walking speed:  Degree of difficulty multiplied with score weight (estimated walking-speed in mph) | Unidirectional | During the past month | Interviewer administered | Assessment of the degree of difficulty in walking a defined distance and speed in PAD patients (s) | No |
| The FAST Functional Performance Inventory | Rejeski, 1995 | Patients with knee osteoarthritis | 5 | Basic ADLs,  Complex ADLs,  Ambulation & climbing,  Transfer,  Upper extremity | Activities of daily living (2)  Activities of daily living (2)  PA related mobility (6)  PA related mobility (6)  PA related mobility (6) | 23 | 6-point Likert type scale | 1 = Usually did with no difficulty, 5 = unable to do, 6 = Usually did not do for other reason | Mean calculated for each domain | Unidirectional | Unclear | Self-administered | Assessment of the ability to perform activities of daily living with or without difficulties in patients with knee osteoarthritis (q) | No |
| The Self-Efficacy for Exercise Scale (SEE) | Resnick 2000 | older adults | 1 | No labelling |  | 9 | 11-point Likert type scale | 0=not confident  10=very confident | Sum of score divided by number of responses | Unidirectional | No recall period | Interviewer-administered | Assessment of self-efficacy for ability to walk/exercise for 20 minutes three times per week in various circumstances in older adults (q) | No |
| The Physical Activity and Disability Survey (PADS) | Rimmer 2001 | Persons with disabilities and chronic health conditions | 3 | Exercise,  Leisure time physical activity,  Household activity | Exercise PA (4)  Leisure PA (7)  Household PA (8) | 11 (of 31) | Categorical (2)  3-point scale  free space (to list activities and the time spent in these listed activities) | Categorical:  yes/no  3-point scale:  1=less than 6 hours a day  2= 6 to 10 hours a day  3=more than 10 hours a day  free space (to list activities and the time spent in these listed activities, time in min/day and day/week) | Not reported | Unidirectional | No recall period | Primarily designed as:  interviewer-administered  can be used as a questionnaire that is  self-administered | Assessment of the performance of physical activity and exercise and the time in listed activities in chronic patients (q) | No |
| Disability Questionnaire | Roland, 1983 | Back pain patients | 1 | Disability | PA limitation (11) | 24 | Categorical | Patients tick if statement applies | Sum | N/A | Today | Self-administered | Assessment of limitations with normal daily activities due to back pain (q) | No |
| The RAYS scale | Rotstein 2000 | Multiple sclerosis patients | 1 (of 3) | Physical functioning (Psychological functioning, social functioning) | Generic PA (1) | 7 (of 50) | 5-point Likert scale | 0=none  1=mild  2=moderate  3=severe  4=extreme | Total score | Unidirectional | Last 7 days | Self-administered | Assessment of the level of problems or difficulties in physical function caused by MS (s) | No |
| The Cancer Rehabilitation Evaluation System (CARES) | Schag 1990 | Patients with cancer | 1 [3 subscales] (of 5) | Domain: Physical [with subscales Ambulation, Recreational Activities, Activities of daily living]  (Psychosocial, medical interaction, marital, sexual) | Generic PA (1) | 12 (of 142) | 5-point-Likert-type scale | 0= not at all (no problem)  4=very much (severe problem) | Total number of endorsed problems  Total severity rating  Average severity rating  Global CIPS score | Unidirectional | Past month | Self-administered | Assessment o the severity of difficulties / problems in performing activities in cancer patients (s) | No |
| Questionnaire of Functional Ability | Schultz-Larsen 1992 | Elderly | 2 | Mobility function,  Lower limb function | PA related mobility (6)  PA related mobility (6) | 33 | Categorical | Yes/no | Not reported | N/A | No recall period | Interviewer-administered | Assessment of the ability to perform physical activities of daily living in the elderly(q) | No |
| Modified Barthel Index | Shah 1989 | Individuals who require assistance of some nature to perform tasks | 9 (of 11) | Personal hygiene, Bathing self, Feeding, On and off the toilet, Stairs, Dressing, Chair/bed transfers, Ambulation, wheelchair management [=alternative to Ambulation] (Bowels, Bladder) | PA relating to self (5)  PA relating to self (5)  PA relating to self (5)  PA related mobility (6)  PA related mobility (6)  PA relating to self (5)  PA related mobility (6)  PA related mobility (6)  PA related mobility (6) | 40 (of 50) | 5-point-Likert type scale | 1=Unable to perform task  5=Fully independent | Sum of weighted items | Unidirectional | No recall period | Interviewer administered, self-administered, telephone administered | Assessment of the degree of dependence which represents the amount of assistance required in functional independence in each item (s) | No |
| The 15-D Measure of HRQOL | Sintonen, 1994, 2001 | Various patient groups | 4 (of 15) | Mobility,  Breathing,  Usual activities,  Sexual activity (Vision, Hearing, Sleeping, Eating, Speech, Elimination, Mental function, Discomfort & symptoms, Depression, Distress, and Vitality) | PA related mobility (6)  Dyspnoea and Symptom related PA (3)  Generic PA (1)  Social PA (9) | 20 (of 75) | Categorical | Patients tick if statement applies | A set of utility or preference weights, elicited from the general public through a 3-stage valuation procedure, is used in an additive aggregation formula to generate the 15D score (single index number) over all the dimensions. The maximum score is 1 (no problems on any dimension) and the minimum score is 0 (being dead). | N/A | Present | Self-administered (can also be interviewer-administered | Assessment of the ability to perform and shortness of breath during activities (s) | No |
| Activities of Daily Living Inventory (ADLI) | So 2008 | Patients with COPD | 6 | Physical stamina, Speech- related,  Sudden change of posture,  Arm & trunk movement, Coordinated dexterity, Prolonged continued exercise | Exercise PA (4)  Other (12)  PA related mobility (6)  PA related mobility (6)  PA related mobility (6)  Exercise PA (4) | 26 | 10 point Likert scale with anchors only defined as well as separate Unable to Perform | 1 = no difficulty 10 = extremely difficult + Unable to Perform | not reported (Presumably sum) | Unidirectional | No recall period | Self-administered | Assessment of difficulty in performing activities of daily living in COPD patients (q) | No |
| The LSA Physical Activity Questionnaire (LAPAQ) | Stel 2004 | Older people | 1 | Physical activity | Generic PA (1) | 7 | Frequency (number) and duration (number) of the activities | N/A | Depending on research question:  The total time in  minutes per day can be calculated by multiplying the frequency  and duration of the individual activities in the previous  2 weeks, summing these values across activities, and dividing by 14. An intensity weighting score can be calculated by multiplying the total  time with the MET score  of each activity, and summing  these values across activities | N/A | During the previous 2 weeks | Interviewer administered | Assessment of the frequency and duration of various physical activities in older people (q) | Yes |
| CHAMPS Physical Activity Questionnaire | Stewart, 2001 | Elderly | 1 | Not reported |  | 123 | Categorical  6-point Likert type scale | Categorical: yes/no  Likert: less than one hour – 9 or more hours | Sum | Categorical: N/A  Likert: unidirectional | Past 4 weeks | Self-administered (can also be interviewer-administered | Assessment of the performance, duration and frequency of various physical activity typically undertaken by the elderly (q) | No |
| Falls Efficacy Scale (FES) | Tinetti 1990 | Elderly | 1 | Falls efficacy | PA relating to self (5) | 10 | 10-point Likert type scale | 1 = extreme confidence, 10 = no confidence at all | Sum of scores on each of the 10 activities. Possible scores range from 10-100 | Unidirectional | No recall period | Interviewer administered | Assessment of self-efficacy for performing several activities of daily living without falling of elderly people (q) | No |
| Rapid Assessment of Physical Activity (RAPA) | Topolski et al., 2006 | Older adults (50 years and older) | 2 | RAPA 1: Aerobic (level and intensity of physical activity)  RAPA 2: Strength & flexibility | Exercise PA (4)  Exercise PA (4) | 9 | Categorical | 0 = no, 1 = yes | RAPA 1: Sum of first 7 items, categorisation into one of five levels of PA (sedentary, under-active, under-active regular, active); RAPA 2: additional items: strength training (1) and flexibility (2), or both (3) | Unidirectional | No recall period | Self-administered | Assessment of the amount and intensity of physical activity in an ascending order in older adults. (q) | No |
| The Seattle Obstructive Lung Disease Questionnaire (SOLQ) | Tu 1997 | Patients with COPD | 1 (of 4) | Physical function (emotional function, coping skills, treatment satisfaction) | Generic PA (1) | 16 (of 29) | 5-point-Likert-type scale | Various PA-Anchors:  1=extremely limited  5=not at all limited or  1=Not at All 5=Severely or  1=Not at All, 5=I couldn’t do activities at All | Responses to the questions of a scale are summed into a raw score, and then transformed to a normalized score ranging from 0 to 100. | Unidirectional | Items 5-16: no recall period  items 17-20: during the past 4 weeks | Self-administered | Assessment of degree of dyspnoea and extent of physical limitation due to lung diseases in performing activities in COPD patients (s) | No |
| The McMaster Toronto Arthritis (MACTAR) Patient Preference Disability Questionnaire | Tugwell 1987 | Patients with rheumatoid arthritis | 2 | Baseline,  Follow-up | Other (12)  Other (12) | 6 | Free report (list activities)  Tick if it applies  2 different categorical scales | Free report (list activities): N/A  Tick if it applies  Categorical 1: yes / no  Categorical 2: Improved / become worse | Multi-item index scores were computed for each patient using the sum of the item responses | N/A | No recall period | Interviewer-administered | Assessment of the impact of arthritis on the ability to carry out selected activities in arthritis (q) | No |
| The Clinical COPD Questionnaire (CCQ) | van der Molen 2003 | Patients with COPD | 1 (of 3) | Functional state (symptoms, mental state) | Generic PA (1) | 4 (of 10) | 7-point Likert type scale | 0=not limited at all  6=totally limited / or unable to do | Sum of PA score / number of PA items | Unidirectional | During the past week | Self-administered | Assessment of limitations in performing activities because of breathing problems in COPD patients (s) | No |
| Questionnaire for Physical Activity Decline in pain (PAD) | Verbunt 2008 | Patients with chronic pain | 1 | Physical activity decline | PA limitation (11) | 20 | First: 5-point-Likert type scale  Afterwards: Categorical | 5-point-Likert type scale:  0=Never, 1=Seldom (1-2 x times per week), 2=Occasionally (3-4 x times per week), 3=Often (5-6 x times per week)  4=Very often (Daily)  Categorical: yes/no | Total sum score | Unidirectional | Last 2 weeks | Self-administered | Assessment of the likelihood of performing various daily activities depending on pain in patients with chronic pain (q) | No |
| Physical activity questionnaire | Voorrips 1991 | Elderly | 3 | Household activities,  Sport activities,  Leisure time activities | Household PA (8)  Exercise PA (4)  Leisure PA (7) | 12 | Household activities: 4- and 5-point scales  sports & leisure time activities: declaring type of activities, hours per week, period of the year | Several different anchors | Household score: sum of item scores divided by number of items  Sport and leisure time scores: calculation of score with use of intensity code for each activity  Questionnaire score (household + sport + leisure time activity score) | Unidirectional | No recall period + for sport and leisure patients need recall hours per week and year | Interviewer administered | Assessment of the frequency of habitual physical activity in order to classify elderly subjects into categories of high, medium and low physical activity in the elderly (q) | No |
| The Physical Activity Scale for the Elderly (PASE) | Washburn 1993 | Elderly | 1 | Physical activities | Generic PA (1) | 12 | First 6 items: Amount of hours per day spent in a specific activity  Following 6 items:  Categorical | Amount of hours per day: N/A  Categorical:  0=did not engage in that activity  1=engaged in that activity | Total PASE score: by multiplying the amount of time spent in each activity (hours a day) by the respective weights. Weights were derived empirically. Further: Summing over all activities | N/A | 7-day period | Self- and interviewer-administered | Assessment of the amount of hours spent and the amount of engagement in activities commonly engaged by older persons | Yes |
| The Pulmonary Functional Status Scale (PFSS) | Weaver 1998 | Patients with COPD | 2 (of 3) | Daily activities/social functioning,  Sexual functioning (Psychological Functioning) | Social PA (9)  Social PA (9) | 25 (of 35) | Various, 4-5 and 6-point scales, mostly: 4-point scale + option to mark 1) “Cannot do this activity because of lung problem” or 2) “Cannot do this because of other health problems” | Various, mostly 4-point scale: 1=extreme difficulty, 4=no difficulty | Mean weighted item score for each subscale. Summing subscale scores for total score | Unidirectional | No recall period | Self-administered | Assessment of the level of difficulty because of lung problems and the ability to perform daily activities in COPD patients (s) | No |
| The COPD Self-Efficacy Scale (CSES) | Wigal 1991 | Individuals with COPD | 1 (of 5) | Physical exertion (Negative Affect, Intense Emotional Arousal, Weather/Environment, Behavioural Risk Factors) | Exercise PA (4) | 5 (of 34) | 5-point Likert scale | A=Very confident  B=Pretty confident  C=Somewhat confident  D=Not very confident  E=Not at all confident | Not reported | Unidirectional | No recall period | Self-administered | Assessment of self-efficacy for avoiding breathing difficulty while performing certain activities in COPD patients (s) | No |
| The Severe Respiratory Insufficiency Questionnaire (SRIQ) | Windisch, 2003 | Patients receiving home mechanical ventilation (HMV) who suffer from advanced respiratory failure due to various underlying diseases | 2 (of 7) | Physical functioning,  Social functioning (Respiratory Complaints, Attendant Symptoms and Sleep, Social Relationships, Anxiety and Psychological Wellbeing.) | Generic PA (1)  Social PA (9) | 14 (of 49) | 5-point Likert scale | -2 = completely untrue, 2 = always true | Sum (higher scores = better HRQoL) | Bi-directional | Last week | Self-administered (can also be interviewer-administered | Assessment of the accuracy of statements describing physical and social situations in advanced respiratory failure patients receiving HMV (s) | No |
| The Manchester Respiratory Activities of Daily Living Questionnaire (MRADL) | Yohannes 2000 | Older patients with COPD | 4 | Mobility,  Kitchen activities,  Domestic tasks,  Leisure activities | PA related mobility (6)  Household PA (8)  Household PA (8)  Leisure PA (7) | 21 | 4-point scale | 3 different anchors: first part:  0=Not at all, 1=with help  2=Alone with help, 3=Alone easily  Second Part:  0=Much more slowly, 1=Quite lot more slowly,  2=A little more slowly, 3=Not at all more slowly  Third Part:  0=Most of the night, 1=For 1-2 hours,  2=For up to 1/2 hours, 3=Not at all | Total score (sum)  1=”alone with difficulty”, “alone easily”, “a little more slowly”, “for ½ hour”, “not at all more slowly”  0=”with help”, “much more slowly”, “quite a lot more slowly”, “most of the night”, “for 1-2 hours” | Unidirectional | No recall period | Self-administered | Assessment of the ability to perform physical activities of daily life alone or with help in older COPD patients (q) | No |
| The Activity of Daily Living Dyspnoea scale (ADL-D scale) | Yoza 2009 | Patients with COPD | 1 | Activities of daily living | Activities of daily living (2) | 15 | 5-point-Likert type scale | 0=Maximally severe  4=Not at all | Total score | Unidirectional | During the past week | Self-administered | Assessment of the degree of breathlessness while performing ADL in COPD patients (q) | No |
| The Quality of Life Questionnaire for Patients With Chronic Respiratory Disease (CV-PERC) | Zaragoza 2009 | Patients with chronic respiratory disease | 3 (of 7) | Physical function,  Sexual function,  Work function (Perceived health and well being, Psychological/emotional function, Cognitive function, Social function) | Generic PA (1)  Social PA (9)  Work PA (10) | 27 (of 50) | 4-point scale | 1=No, not at all  4=Yes, definitely | Domain score (total of points of each item in the group) & total overall score (0-150; total of points of all items) | Bidirectional | In the last month | Self-administered or Interviewer-administrated | Assessment of symptoms and their intensity on various aspects of patients’ with chronic respiratory disease life (s) | No |
| Quality of Life Instrument (QOL-Instrument) | Zhou 2009 | Chronic liver disease patients with minimal hepatic encephalopathy | Unknown | Unknown | Unknown | Unknown | Unknown | Unknown | Unknown | Unknown | Unknown | Unknown | Questionnaire was available in Chinese only | Unknown |
| Scale of Older Adults’ Routine (SOAR) | Zisberg 2005 | Older Adults | 4 (of 5) | Basic routine activities,  Instrumental activities,  Leisure activities,  Social participation, (Rest) | Generic PA (1)  Activities of daily living (2)  Leisure PA (7)  Social PA (9) | 39 (of 42) | 1) Number of times (doing this activity) per day and/or per week  2) Time of day (doing this activity) and how long the activity takes in minutes | N/A | Five scores, based on five subscales, as well as a total score on each.  Scoring considers the following components:  endorsement (=number of relevant activities / number of total activities)  frequency, duration, total duration and actual time of activities, additionally a stability score was calculated (SD) | N/A | Yesterday, if yesterday was a typical day. Otherwise the participants are asked to choose another day that was typical of the last seven days | Interviewer administered | Assessment of the time performing routine activities with a diary of older adults (s) | Yes |

1) Yes = Quantifications including METs etc, No = scale values
